# Diagnosis and treatment of patients with gastroesophageal reflux disease – a systematic review of cost-effectiveness and economic burden

**DOI:** 10.1186/s12913-024-11781-8

**Published:** 2024-11-06

**Authors:** Petra Maresova, Lukas Rezny, Jan Hruska, Blanka Klimova, Lee L Swanstrom, Kamil Kuca

**Affiliations:** 1 Betthera s.r.o, Hradec Kralove, Czech Republic; 2 Faculty of Informatics and Management, University of Hradec Kralove, Hradec Kralove, Czech Republic; 3 IHU Strasbourg, Innovat Officer & Sci, Strasbourg, France

**Keywords:** Gastrointestinal diseases, Cost-effectiveness, Gastroesophageal reflux disease, Diagnostic methods

## Abstract

**Background:**

This study aims to review the existing knowledge on the cost-effectiveness and item costs related to the diagnosis and treatment of gastroesophageal reflux disease (GERD) patients at different stages.

**Methods:**

The study adhered to the PRISMA guidelines. The systematic search involved several steps: finding and identifying relevant articles, filtering them according to the set criteria, and examining the final number of selected articles to obtain the primary information. The number of articles published between 2013 and September 2024 in the Web of Science and PubMed databases was considered. The CHEERS checklist was used for the risk of bias assessment. Ultimately, 36 studies were included.

**Results:**

Regarding the cost-effectiveness of GERD treatment, Proton pump inhibitors (PPIs) appeared to be the dominant solution for non-refractory patients. However, this might change with the adoption of the novel drug vonoprazan, which is more effective and cheaper. With advancements in emerging technologies, new diagnostic and screening approaches such as Endosheath, Cytosponge, and combined multichannel intraluminal impedance and pH monitoring catheters should be considered, with potential implications for optimal GERD management strategies.

**Discussion:**

The new diagnostic methods are reliable, safe, and more comfortable than standard procedures. PPIs are commonly used as the first line of treatment for GERD. Surgery, such as magnetic sphincter augmentation or laparoscopic fundoplication, is only recommended for patients with treatment-resistant GERD or severe symptoms.

**Other:**

Advances in emerging technologies for diagnostics and screening may lead to a shift in the entire GERD treatment model, offering less invasive options and potentially improving patients’ quality of life.

**Supplementary Information:**

The online version contains supplementary material available at 10.1186/s12913-024-11781-8.

## Introduction

Gastrointestinal (GI) diseases, ranging from functional problems to cancers, are among the most frequent medical ailments and a major source of morbidity and care costs worldwide [1]. According to United European Gastroenterology, digestive cancers are responsible for over one-third of cancer related deaths. United European Gastroenterology records 332 million prevalent cases and 498 thousand deaths caused by digestive diseases in the year 2019 alone amongst its member countries [2]. Global burden of digestive diseases is substantial and varies markedly according to age, sex, SDI, and geographical region [3]. 

Gastroesophageal reflux disease (GERD) affects an estimated 1.03 billion people worldwide [4]. Moreover, it is extremely costly in terms of treatment costs and patients’ quality of life (QoL) [5]. The overall burden of GERD continued to worsen with the prevalent cases increasing by 77.53% from 441.57 million in 1990 to 783.95 million in 2019 [6]. Furthermore, GERD is associated with several economic and social issues. In the United Kingdom, the estimated cost of healthcare and work absenteeism due to GERD is £760 million, whereas the cost is $24 billion in the United States [4]. In a 2006 study in Germany [7], the reported direct cost (physician visits, costs of drugs, costs of tests, and hospitalization) of GERD per patient per year was approximately €342 (equal to 396 PPP$ or purchasing power parity dollars). Schwenkglenks et al. [8] estimated this to be equal to CHF 185 (equal to 110 PPP$). Over the last 20 years, GERD-associated disabilities have increased globally [6, 9]. Certain factors such as obesity, pregnancy, smoking, certain foods (fatty or fried foods), beverages (alcohol or coffee), and medications (aspirin) increase the risk of GERD. If stomach acid reflux into the lower esophagus continues for a long time, it can cause complications such as esophageal inflammation, stricture, ulceration, perforation, ‘Barrett’s esophagus, and even esophageal adenocarcinoma. Therefore, if GERD is diagnosed early, continuously monitored, and treated appropriately, these complications can be avoided. GERD is not a fatal disease; however, esophageal cancer is fatal, leading to the death of 5.5% of all types of cancer patients [10]. Currently, esophageal pH monitoring and endoscopy can be used to diagnose GERD (and other GI diseases) and its effect on the esophageal mucosa; however, these procedures are invasive, very unpleasant to patients (nose endoscopy), and can miss cases with a fluctuating course. Foroutan et al. [11], in 2017, showed that multichannel Intraluminal Impedance (MII-pH) is particularly effective in distinguishing more reflux episodes. Moreover, MII-pH is more sensitive and specific for diagnosing GERD than endoscopy or pH measurements. In addition, non-catheter-based devices such as wireless pH capsules are popular tools for GERD diagnoses [12]. 

This review aimed to describe the existing and cutting-edge knowledge on cost-effectiveness or item costs related to the diagnosis and treatment of patients with GERD at different stages of the disease, including the possible consequences of the disease in cases of non-early detection. Our objectives were to compare the costs of current solutions with one another and establish a basis for research and progressive development of new diagnostic solutions (e.g., eHealth CAPsule for digestive disease diagnostics and therapy). We believe that such a comparison will provide relevant information for comparing the cost-effectiveness of the new diagnostic solutions and their likelihood to succeed in the market.

## Methods

### Study design

The methodology of this systematic review followed the standard Preferred Reporting Items for Systematic Review and Meta-Analysis (PRISMA) guidelines [13] and the bibliometric analysis by Leung et al. (2017) [14] to discuss the diagnostics and treatment of patients with GERD and examine associated cost-effectiveness outcomes and direct and indirect costs to provide recommendations for research and development (R&D) on improving patient’s QoL and cost-saving measures. For risk of bias assessment, the CHEERS checklist was used, as it is suitable for cost-effectiveness studies.

Two groups of reviewers, Group 1 (Marešová, Hruška) and Group 2 (Režný, Klímová), used a standardized form or data extraction tool to code each paper separately. This was done to reduce potential bias and errors. Both groups collected data on the study’s design, methodology, participants, environment, interventions, and outcomes. In case of any conflicts, Swanstrom Lee made the final decision. The design was overseen by Kamil Kuča. All authors have approved, written, and reviewed the manuscript.

### Search strategies and criteria

#### Information sources and search strategy

This review covers the period from January 2013 to September 2024 because a review paper with a similar focus titled “Economic Evaluations of Gastroesophageal Reflux Disease Medical Management: A Systematic Review” was published in 2014 [5]. The authors of the present review aimed to update the existing knowledge. Additionally, we aimed to include the societal perspectives regarding the costs of GERD, specifically long-term effects and indirect costs. Long-term stomach acid reflux into the lower esophagus leads to complications such as Barrett syndrome and esophageal adenocarcinoma. Studies whose overall design fit the study aim were included with these aspects in mind.

**Fig. 1:**
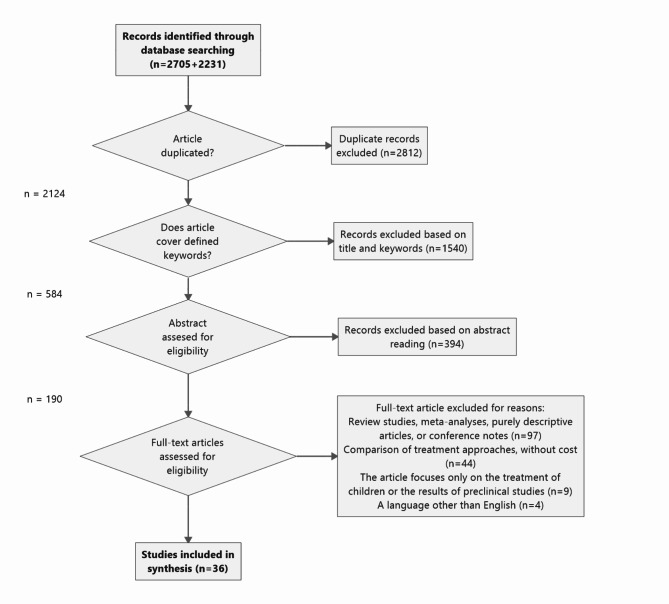
Article filtering procedure: Distribution of the articles (between 2013 and September 2024). Filter – 2013-01-01 to 2024-09-30. Document types: Article or Review Articles

The systematic search consisted of several steps: finding and identifying relevant articles, filtering them according to the set criteria, and examining the final number of selected articles to obtain the primary information. The number of articles published between 2013 and September 2024 found using the keywords “Gastroesophageal reflux disease,” “Cost,” “Technology,” “Capsule,” and “Proton Pump Inhibitors” and their combinations are shown in Annex 3. Finally, 36 articles were included in the analysis (Fig. 1). A list of included articles is provided also in Annex 1.

#### Design of cost comparability

Cost data for the studies originating from the US were inflation adjusted with the help of Inflation calculator of Federal Reserve Bank of Minneapolis (https://www.minneapolisfed.org/about-us/monetary-policy/inflation-calculator). Studies from different countries were first inflation adjusted based on their national/regional inflation rates using CPI inflation calculators [15, 16] and then were adjusted for purchasing power parity for conversion to USD [17]. So, in the following sections, results are reported in the constant 2023 dollars unless otherwise stated.

#### Eligibility criteria

Only articles in the English language were considered.

#### Inclusion criteria

- Articles published from 2014 to September 2024—inclusive.
- English-written peer-reviewed full-text articles.
- Articles focused on cost-effectiveness outcomes in the diagnosis and treatment of patients with GERD, quality-adjusted life year (QALY), incremental cost-effectiveness ratio (ICER), and ICER/QALY indicators.
- Studies that described direct and indirect costs.
- Studies focused on GERD, the consequences of the disease, follow-up treatment or surgery, and associated costs.
- Studies that described the used therapies, drugs, or surgical procedures in long-term care for people with GERD.
- Studies that aimed to reveal the present state of GERD-related costs and economic burden and its consequences.

#### Exclusion criteria

- Articles published in a language other than English.
- The study was published before 2013.
- Review studies, meta-analyses, purely descriptive articles, or conference notes.
- An article that focused only on the treatment of children or the results of preclinical studies.
- Studies that focused on clinical efficacy with no relation to costs.
- Studies with a comparison of two treatment approaches without reference to costs.
- Studies that discussed diagnostic tools, their comparison, and description of effectiveness but no relation to costs or QALY indicators.
- Studies on the specification of management of the disease in different contexts.
- Studies on extraesophageal reflux and hybrid hiatal hernia repair.

#### Cluster analysis

Cluster analysis was performed to detect the key areas in which cost-effectiveness was addressed in the analyzed domain. These findings highlight the crucial aspects of the treatment model for GERD.

**Fig. 2:**
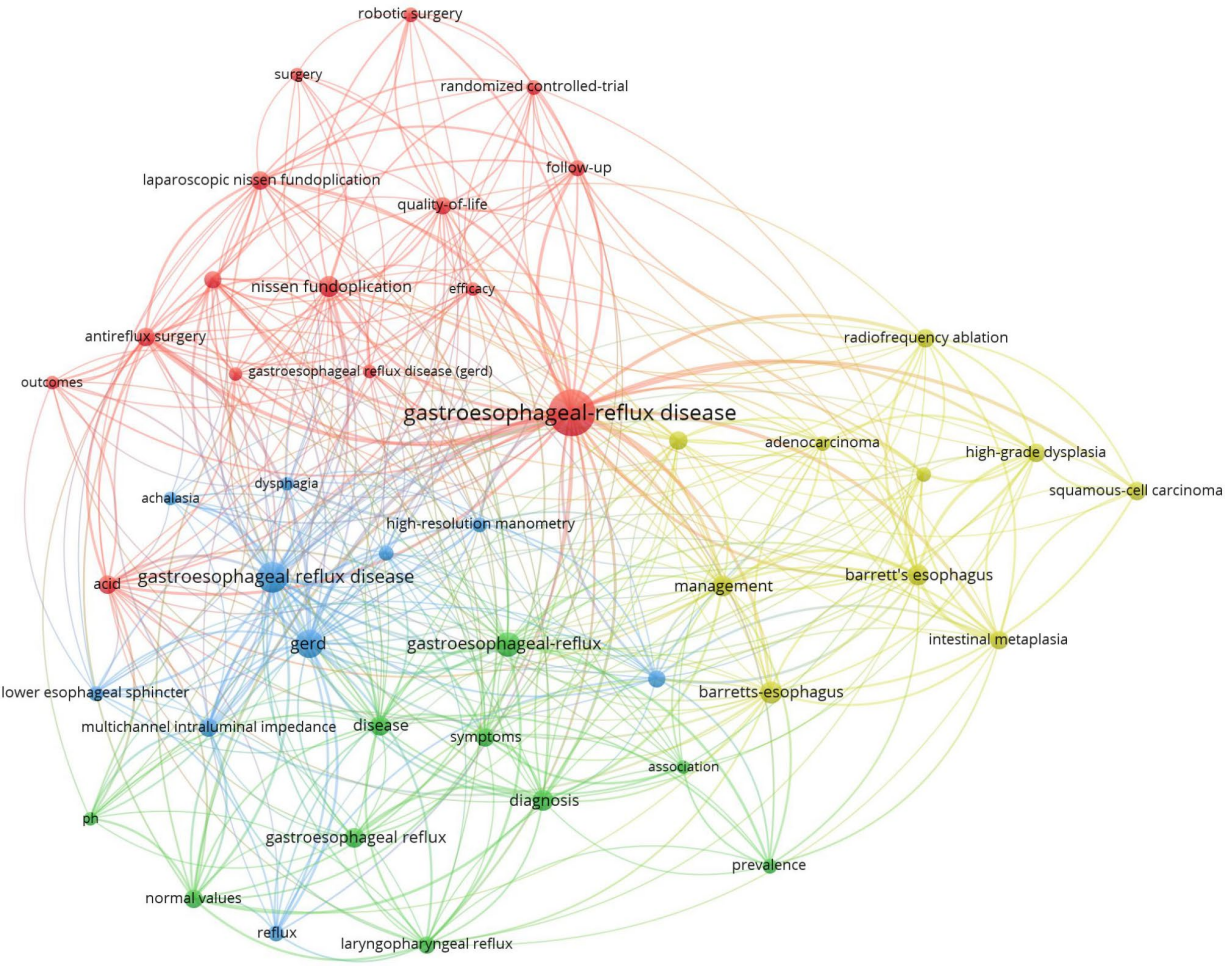
Gastroesophageal Reflux Disease + Technology, Document types: Articles or Review Articles, Articles in search: 163

Figure 2 shows the four clusters in the results. The red cluster is mainly related to the results of clinical trials of different treatment approaches, evaluating efficacy and QoL; the yellow cluster focuses on other likely related diseases, such as Barrett’s esophagus; the green cluster concentrates on general disease characteristics, such as prevalence and symptoms; and the blue cluster focuses on related drugs. The overlaps between the clusters were obvious.

**Fig. 3:**
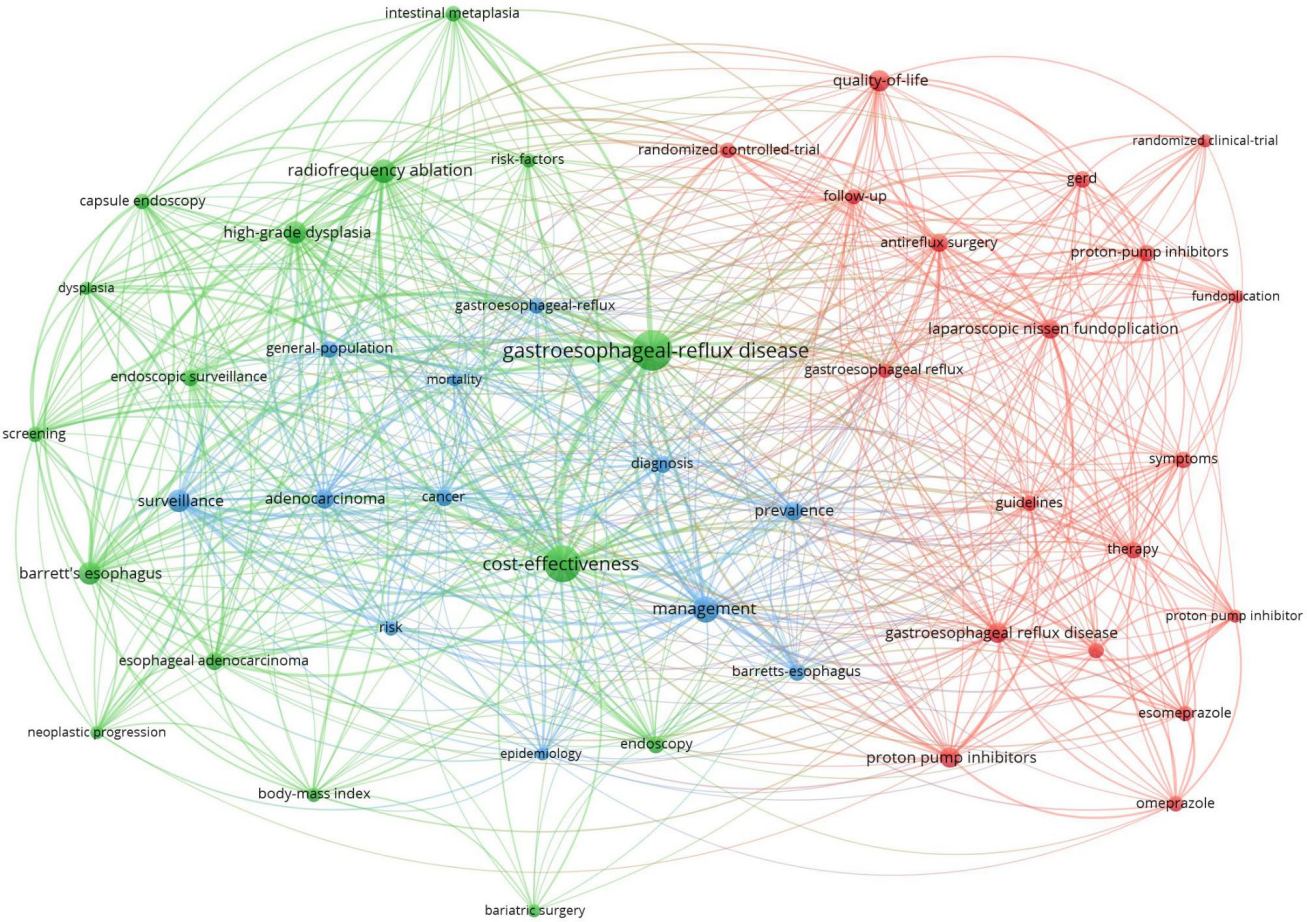
Gastroesophageal Reflux Disease + Cost Effectiveness, Document types: Articles or Review Articles, Date: Last 10 years, Articles in search: 119

The next cluster (Fig. 3) was related to the main keyword, “Cost Effectiveness.” In this regard, the most significant was the green cluster, which focuses directly on the cost-effectiveness of diagnosis, treatment, and care. It highlights the following areas in GERD: screening, endoscopy, bariatric surgery, and, in terms of health context, dysplasia, Barrett’s esophagus, and cancer. Meanwhile, the red cluster detects specific approaches in therapy in relation to the symptoms while considering the results of clinical trials.

Variables were defined based on the above-described thematic clusters, which were derived from the set keywords. Variables characterize the economic context (cost-effectiveness and item costs) in the context of the methodological approaches used. Variables are applicable across clusters; that is, the general characteristics of the study are study design and approach, relevant characteristics of the study population, intervention/comparator, country, outcome/cost-effectiveness, and time horizon. Regarding the metrics used in the studies included in the present review, the variables were as follows: modeling approaches/model type, number and type of health conditions/events, cycle length/timeline, assumptions, and handling of uncertainty. The last group of monitored variables was cost items: cost items according to the relevant reimbursement catalog, costs per application, treatment costs, currency, and year.

### Results of the systematic review

In total, 36 studies were included in this systematic review (Annex 1). Most of the studies originated from the United States (18), Korea (4), and Japan (4). Only five studies were conducted in Europe, specifically in the United Kingdom (3) and France (2). One of the studies was a joint study of European and North American scholars, and the other was a joint study of Filipino and Malaysian researchers. One study was conducted in South America and Brazil. The time horizon of these detected studies spanned from 20-hour interval observations to 30 years. Furthermore, the number of participants included in the studies varied, ranging from 1 to a hypothetical cohort of 1,000,000 patients. Table 1 provides an overview of the main characteristics and findings of the selected studies.

**Table 1:** Summary of the detected studies

Study	Study type	Study design and approach	Relevant characteristics of the study population	Strategies		Country	Outcome Cost effectiveness	Time horizon
				Intervention	Comparator			
Lawenko & Lee, 2015	Observational	Indications for Esophageal pH Testing Using the Bravo Capsule System	Asian population with mentioned 32 patients	Optimal threshold cut-off values	Sub-optimal pH values	Philippines and Malaysia	A complete Bravo system costs around $25,704. A single-use Bravo capsule with a delivery device costs $225 compared to the conventional trans-nasal pH catheter which costs $62 (in $2016).	24 h intervals
Afaneh et al., 2016	Cost-effectiveness Analysis	Review of 100 consecutive patients who underwent wireless pH monitoring for suspected GERD	100 consecutive patients who underwent wireless pH monitoring	PPI costs	Procedure costs	USA	Maximum cost-savings occurred in patients with extraesophageal symptoms ($2948–$31,389 per patient).	215 weeks
Sami et al., 2021	Comparative Cost Effectiveness	Markov modeling was performed in 3 scenarios in 50 years old individuals	The model simulated hypothetical cohorts of 500,000 individuals.	GERD-based and GERD-independent testing scenarios	Cost-effectiveness of BE	Europe and North America	Swallowable esophageal cell collection devices with biomarkers were cost-effective (<$35,000/QALY) and were the optimal screening tests in all scenarios.	Time intervals (cycles) of 1 month in length
Benaglia et al., 2013	Health Benefits and Cost Effectiveness	Microsimulation modeling of a hypothetical cohort of 50-year-old men in the United Kingdom	Screening 50-year-old men with symptoms of gastroesophageal reflux disease	Cytosponge and Endoscopy screening	No screening (symptomatic management only)	UK	In a microsimulation model, screening 50-year-old men with symptoms of gastroesophageal reflux disease by Cytosponge is cost-effective and would reduce mortality from esophageal adenocarcinoma compared with no screening.	Duration of the microsimulation cycle (30 days)
Park et al., 2020	Cost-Effectiveness Study	Decision tree and Markov model to obtain the costs and quality-adjusted life years (QALYs) of the surgical and medical strategies.	Patients aged 50 years old who required a continuous double dose of PPIs.	Surgical strategy	PPI medication	Korea	The model predicted that the surgical strategy had a cost savings of $551 and the QALYs had a gain of 1.18 as compared with the medical strategy.	10 years
Park et al., 2020	Cross-sectional analysis	Generalized linear models were used to estimate cost ratios for comparing the medical costs of the surgery and medication groups	86 936 participants in the medication group and 40 in the surgery group.	Medical expenditures	Compare ARS and PPI therapy for the treatment of GERD	Korea	The medical expenditures of the surgery group within 90 days of anti-reflux surgery (ARS) were 16.9-fold higher compared to those of the medication group.	5 years
Pandolfino et al., 2020	Budget impact analysis	An economic budget impact model was developed over a 1-year time horizon that compared current treatment of GERD	Eligible medication-refractory mechanical GERD patients included in the analysis were assumed to be 9,595	Removable magnetic sphincter augmentation (MSA)	laparoscopic Nissen fundoplication (LNF)	USA	Base-case analysis estimated a net cost savings of $111,367 with the introduction of the MSA.	1-year time horizon
Park et al., 2020	Comparison study	Compare the clinical characteristics, medical utilization, and medical costs of anti-reflux surgery and proton pump inhibitor (PPI) treatments.	Korean patients who underwent fundoplication (*n* = 342, surgery group) (*n* = 130987, medication group)	ARS and PPI treatment	Laparoscopic anti-reflux surgery	Korea	The average cost of fundoplication was $4,631. The costs of GERD treatment in the first year after surgery and during the follow-up period were $78.1 and $50.1 per month, respectively ($2020).	10 years
Azzam et al., 2021	Comparison and monitoring study	twenty-five patients with symptoms of gastroesophageal reflux were prospectively submitted, in a simultaneous initial period, to 24-hour catheter esophageal pH monitoring and 4a 8-hour wireless system.	Twenty-five patients with symptoms of gastroesophageal reflux	The capsule	The catheter	Brazil	Regarding the expenses, the capsule (single use) costs $411.53, and the catheter (reused five times) $39.22; so, the catheter costs only $7.84 per use ($2021).	48-hour wireless monitoring cycles
Park et al., 2013	Cost efficiency and evaluation analysis, cost-benefit analysis	randomized, open-label study enrolling 279 patients with erosive esophagitis A or B (Los Angeles classification) and typical gastroesophageal reflux disease symptoms.	279 patients with erosive esophagitis A or B	Omeprazole	Rabeprazole	Korea	By the cost-minimization analysis, the mean total costs per patient for remaining symptom-free for 6 months were 241,775 won ($357) for omeprazole and 287,115 won ($425) for rabeprazole, respectively.	42 weeks
Yokoya et al., 2019	Cost-utility analysis	A Markov simulation model was developed to evaluate the cost-effectiveness of vonoprazan-first, esomeprazole-first, and rabeprazole-first strategies	Japanese clinical trial data for vonoprazan versus lansoprazole as healing and maintenance treatment	‘vonoprazan-first’ strategy	‘esomeprazole- or rabeprazole-first’	Japan	Expected costs of the vonoprazan-, esomeprazole-, and rabeprazole-first strategies were ¥36,194 ($402), ¥76,719 ($853), and ¥41,105 ($457), respectively, over 5 years.	5 years (4- week cycles).
Funk et al., 2015	Long-term cost-effectiveness analysis	A Markov model was generated from the payer’s perspective using a 6-month cycle and 30-year time horizon. The base-case patient was a 45-year-old man with symptomatic GERD taking 20 mg of omeprazole twice daily.	The base-case patient was a 45-year-old man	Endoscopic and surgical Management cost	PPI cost	USA	Low-cost PPIs, Stretta, and laparoscopic Nissen fundoplication all represent cost-effective treatment strategies. In this model, when PPIs exceed $90 per month, medical therapy is no longer cost effective.	6-month cycle and 30-year time horizon
Heberle et al., 2017	Cost-Effectiveness analysis	Simulated the effect of a 1-time screen for BE in male patients with GERD, 60 years of age,	1,000 patients	ICERs for cytosponge screening	No screening	USA	The ICERs for cytosponge screening compared to no screening ranged from $28,791 to $33,307. For screening patients by endoscopy compared to cytosponge, the ICERs ranged from $143,041 to $330,361 in constant 2017 dollars.	5 years
Miwa et al., 2016	Medical and treatment analysis	Longitudinal analysis among newly diagnosed GERD patients	An insurance claims database with data on approximately 1.9 million company employees	Medical costs for patients with GERD	Type 2 diabetes, hyperlipidemia, and hypertension	Japan	The mean medical cost PPPM for GERD patients aged 20–59 was JPY 31,900 ($361.56), which was approximately 2.4 times the mean national health care cost PPPM for Japanese people aged 20–59 in 2013 (JPY 13,500 = $153).	10 years
Kleiman et al., 2014	Cost-Effective analysis	A cohort of 100 consecutive patients who underwent 24-h esophageal pH monitoring	100 patients	Esophageal pH Monitoring	Empiric Trials of Proton-Pump Inhibitors	New York, USA	If the sensitivity of pH monitoring was 96%, early referral for pH monitoring would have saved between $1,197 and $6,303 constant 2013 dollars per patient over 10 years.	7 years
Habu, 2019	Treatment comparison	Cost-effectiveness Analysis	Simulation	Potassium-competitive acid blocker (P-CAB), Vonoprazan	PPI, Lansoprazole	Japan	Cost-effectiveness ratio (Yen/Disease-free day); Results: 58 Yen/day ($0.64) for P-CAB vs. 68 ($0.76) for PPI	1 year
Habu et al., 2021	Treatment comparison	Cost-effectiveness Analysis	Simulation	P-CAB (Vonoprazan)	PPI, Lansoprazole	Japan	Cost-effectiveness ratio (Yen/Disease-free day); Results: Intermittent P-CAB strategy 31 Yen/day ($0.35), Intermittent PPI strategy 39 Yen/day ($0.43).	1 year
Bruley Des Varannes et al., 2013	Observational	Prospective, multicenter, observational study. Work productivity loss was assessed using the WPA-GERD questionnaire.	716 patients (mean age: 46.3 years)	-	-	France	Mean associated cost per patient/week; Results: 313 EUR ($723).	1 year
Howden et al., 2021	Observational	Retrospective analysis of the IBM MarketScan databases	399,017 GERD patients, 103,654 with refractory symptoms	-	-	USA	Health care costs per patient per year; $26,057 for patients with refractory GERD, $15,285 without in constant 2021 dollars.	1 year
Ayazi et al., 2020	Observational	Direct costs calculation	Patients who underwent MSA over a 2-year period, 195 patients who underwent MSA and 1131 that had LNF	Magnetic sphincter augmentation (MSA) surgery treatment	Nissen fundoplication (LNF)	USA	Treatment cost per member per month (reimbursement); MSA treatment $ 305 PMPM prior to surgery and $ 104 PMPM after surgery, $ 233 PMPM and $126 PMPM for LNF in constant 2019 dollars.	2 years
Singer & Smith, 2021	Cost-effectiveness Analysis	A decision analytic model	The reference case was a 60-year-old white male with GERD	Wide Area Transepithelial Sampling with Computer‑Assisted Analysis (WATS3D) with FB	random 4-quadrant forceps biopsies (FB, “Seattle protocol”)	USA	Screening with WATS3D costs an additional $1,219 and produced an additional 0.017 QALYs, for an ICER of $71,395/QALY, all expressed in 2020 constant USD.	Unspecified
Gronnier et al., 2014	Observational	Health outcomes, quality of life and cost-analysis assessment	Patients who underwent a primary LF for symptomatic uncomplicated gastroesophageal reflux disease in University Hospital Claude Huriez *n* = 292.	Day-case laparoscopic Nissen-Rossetti fundoplication (LF)	Inpatient LF	France	Estimated direct healthcare costs per patient were 2,248 euros ($5,170) in the day-case group vs. 6,569 euros ($15,109) in the inpatient group.	Cases from 2004 to 2011
Lai et al., 2022	A quasi-experimental study	The study was conducted at a call center pharmacists provided MTM services to patients telephonically Providing conversion recommendations lower-cost PPIs.	Adult patients aged 18 years who received higher-cost PPIs were included, *n* = 40	Lower-cost PPI	Higher-cost PPI	USA	The total cost avoidance from medication conversions per patient per year $; $4,485.6.	1 year
Kleppe et al., 2020	Observational	The study evaluates healthcare utilization during the 90-day post-operative period following ARS including fundoplication and/or paraesophageal hernia (PEH) repair	A total of 40,853 patients were included from Truven Health MarketScan^®^ Database who underwent ARS (Anti-reflux surgery)	-	-	USA	Direct costs; The mean cost of the index surgical admission was $24,034. Patients requiring one or more related surgical readmissions accrued additional costs of $29,513 in 2020 constant dollars.	90 days
Sharaiha et al., 2014	Cost-effectiveness Analysis	Markov model	A hypothetical cohort of 50-year old white men with Barrett’s esophagus, *n* = 250,000	chemoprevention with PPIs	No chemoprevention	USA	Administration of PPIs cost $23,000 per patient resulted in a gain of 0.32 QALYs for an incremental cost-effectiveness ratio of $12,000/QALY (2014 constant USD).	30-years
Owen et al., 2014	Treatment comparison	a cost and health outcomes analysis	*n* = 12,079 patients receiving fundoplication	robot-assisted laparoscopic fundoplications (RLF)	Open (OLF) and Conventional Fundoplication (CLF)	USA	Direct costs of surgery: CLF=$7,968; RLF = $10,644; OLF = $12,766; 2014 constant USD.	-
Schlottmann et al., 2017	Treatment comparison	a cost and health outcomes,	The study included adult patients (18 years and older) diagnosed with gastroesophageal reflux disease (GERD), who underwent either laparoscopic or open fundoplication, *n* = 75,544	Laparoscopic anti-reflux surgery (LARS)	open anti-reflux surgery (OARS)	USA	the laparoscopic approach reduced the length of stay by 2.1 days, and decreased hospital charges by $9,530 in 2017 dollar prices.	-
Furneri et al., 2019	Diagnostic comparison	decision tree / Markov model; Budget impact analysis and cost-effectiveness analyses	Simulated cohort of BE patients (*n* = 161,657 at Year 1; estimated annual increase: +20%)	opto-digital chromoendoscopy with the use of narrow-band imaging (NBI)	high-definition white light endoscopy (HD-WLE)	UK	Total cost savings (British pound); adoption of NBI resulted in a cost reduction of £458.0 ($863.86) mln.	7 years
Yang et al., 2015	Diagnostic comparison	estimated the impact of surveillance endoscopy for BE, cost-effectiveness analysis	among the modeled Western population	adequate surveillance (AS)	inadequate surveillance (IAS), and no surveillance groups	USA	incremental cost-effectiveness ratio (constant 2015 EUR/% Two-year disease-specific survival percentage of esophageal adenocarcinoma; Adequate Surveillance patient group had lower incremental cost-effectiveness ratio (6,116 €/% vs. 118,347 €/%) than Inadequate S. group (6,187.80€/% vs. 119,736.39€/%).	5 years
Moriarty et al., 2018	Diagnostics method comparison	comparative effectiveness randomized trial; accounting for (direct medical + indirect costs	209 patients were screened (61 sEGD, 72 huTNE and 76 muTNE), Olmsted County, Minnesota residents 50 years of age or older	unsedated transnasal endoscopy (uTNE)	sedated endoscopy (sEGD)	USA	Cost of screening; One-month total sum of direct + indirect costs of screening; sEGD $2,149, Hospital uTNE $976.38 (constant 2018 USD).	30 days
McCarty et al., 2022	Cost-effectiveness Analysis	Decision-analytic Markov cohort model	Cohort consisted of patients aged 50 years	Transoral incisionless fundoplication (TIF 2.0)	Omeprazole 20 mg twice daily, laparoscopic Nissen fundoplication [LNF]	USA	One-way sensitivity and threshold analyses showed TIF 2.0 remained cost-effective up to a total procedural cost of $ 11,724.94 among patients on twice-daily 20-mg omeprazole.	10 years
Harper et al., 2023	Cost-effectiveness Analysis	Analytical framework used to assess cost-effectiveness of RefluxStop was a state transition (Markov) model	1,000 patients whose starting age was 52 years and 56% were male	Novel implantable device (RefluxStop)	PPI-based medical management (MM), LNF and magnetic sphincter augmentation (MSA, LINX system)	UK	The results of the cost-effectiveness analysis demonstrated that RefluxStop is highly likely to be a cost-effective treatment option for GERD patients when compared with treatment options currently available within NHS England and Wales.	Model cycle length was 1 month
Swart et al., 2021	Cost-effectiveness Analysis	A Markov model	Individuals aged 50 years and over. 6,834 patients were enrolled, 1750 were eligible.	Cytosponge screening	No screening	England	Per person, one round of Cytosponge-TFF3 screening, including confirmatory endoscopy and treatment, in the intervention arm costed £82 ($149.5) more than usual care and generated an additional 0.015 quality-adjusted lifeyears (QALYs) at an ICER of £5,500 per QALY gained.	One-year cycle-length and a lifetime time horizon
Honing et al., 2019	Cost-effectiveness Analysis	A Markov model	50-year-old white men. simulated 10,000 patients.	uTNE or standard endoscopy	No screening	USA	Costs of uTNE, standard endoscopy, and no screening were estimated at, $2,495, $2,957, and $1,436, respectively in constant 2018 USD.	Lifetime horizon
Törer & Aytaç, 2017	Cost Analysis	Retrospective cross-sectional descriptive study	102 patients with suspected non-erosive GERD and underwent 24 h impedance/pH-monitoring.	MII/pH monitoring	Conventional pH monitoring	Turkey	The cost of the single- step algorithm using MII catheter was calculated as $15,300, while the total cost of two-step scenario would have been predicted as $16,890 in 2016 constant prices.	24 h intervals
Harper et al., 2024	Cost-effectiveness Analysis	A Markov model	Group of 1,000 patients with a lower age limit of 52 years, composed of 56% males.	RefluxStop	PPIs, Nissen fundoplication, and MSA	Switzerland	Higher QALYs and lower costs were provided by RefluxStop compared to Nissen fundoplication and the MSA system.	Model cycle length was 1 month

Exchange rate for price conversions as of July 26, 2022 using https://www.bankofamerica.com/foreign-exchange/exchange-rates/

Most studies (12) have focused on the theoretical modeling of cost-effectiveness using Markov models. This is because these models can predict the future states of the healthcare system in terms of determining the possible costs for a certain medical device or change of the treatment protocol. Observational studies were included among the remaining articles. They usually report the total costs obtained by the authors or, in some instances, only the costs of the selected novel diagnostic method (Table 1).

In the search phase, certain studies initially appeared to meet the criteria for inclusion in the final selection of articles. However, they were subsequently excluded, primarily due to insufficient descriptions of costs [18, 19].

With regard to cost-effectiveness, only three studies reported expenditure estimates for the general treatment of GERD. Eight studies reported proton pump inhibitor (PPI) administration and treatment optimization and compared them with other medications. Six studies focused on anti-reflux surgery (ARS) costs and various in-between surgery cost comparisons. Four studies compared the overall cost-effectiveness of PPI treatments, surgeries, and endoscopic treatment methods. At present, diagnostic and screening methods are the most popular considerations in the field of economic evaluation of GERD treatment, as 11 articles were identified in this area. Three studies evaluated the Bravo capsule for pH monitoring in GERD diagnostics, whereas the rest of the articles focused on Barrett’s esophagus diagnostic methods and screening procedure effectiveness. More details about studies are in Table 1.

Overall, the contents of the studies can be divided into the following areas: a description of the general economic burden of GERD, including direct and indirect costs; a description of the costs of PPI therapies, followed by the costs of surgeries and their comparisons; and description of the costs of diagnostic and screening methods for GERD, Barrett’s esophagus, and adenocarcinoma. Before presenting the cost data, it is necessary to clarify the current GERD treatment guidelines. In patients experiencing relief during the initial 8–12 week PPI therapy, an attempt to discontinue medication should be considered. If necessary, these patients can be treated again with intermittent PPI therapy. If a patient does not experience satisfactory relief from their symptoms or no relief at all, it is recommended to try to optimize their PPI therapy for a period of 2–4 weeks. If this attempt does not succeed, the patient should be diagnosed with esophagogastroduodenoscopy and/or impedance-pH monitoring based on their observed symptoms. Then, depending on the results obtained, a decision can be made regarding the next suitable treatment steps, which could be escalated medical therapy or surgical/endoscopic intervention [20].

Therefore, PPIs can be considered first-line treatment, while surgical/endoscopic interventions can be considered as second-line treatment options that may be effective if PPIs are unsuccessful. A detailed discussion of the findings is provided below.

#### Costs of treatment of patients with GERD

Table 2 provides an overview of the economic burden of treating patients with GERD, considering the selected treatment methods and solution type.

**Table 2:** Costs of the intervention and comparator(s)

Study	cost item according to relevant reimbursement catalogue	Costs per application [Original currency unit (2023 constant US dollars)]	Treatment	Treatment cost	Currency	Year
Lawenko & Lee, 2015	Bravo capsule	$225 ($289.25)	Diagnostic tool	$25,704 ($33,044)	USD	2015
	pH catheter	$62 ($79.71)				
Afaneh et al., 2016	BRAVO wireless pH monitoring	$614 ($789)	Procedure/diagnostic – monitoring tool		USD	2015
	Esophagogastroduodenoscopy	$400 ($514)				
	Total BRAVO + Esophagogastroduodenoscopy	$1,014 ($1304)				
	Catheter-based pH monitoring	$340 ($437)				
	Esophageal manometry	$350 ($450)				
Benaglia et al., 2013	An excess inpatient stay day cost	$268.20 ($350.80)	Patient care		USD	2013
	Postsurgery follow-up – 2 outpatient visits/y	$392.67 ($513.60)	Patient care			
	Endotherapy (RFA EMR)	$1,725.20 ($2,256.52)	Diagnostic tool			
	Endoscopy biopsy (screening/ surveillance)	$785.84 ($1,027.47)	Diagnostic tool			
	Cytosponge screening	$152 ($198.81)	Diagnostic tool			
Park et al., 2020	Medication	$10,247 ($12,064)	PPIs, including lansoprazole, dexlansoprazole, omeprazole, pantoprazole, s-pantoprazole, rabeprazole, ilaprazole, or esomeprazole		USD	2020
	Surgery	$9,696 ($11,415)	Surgery			
Park et al., 2020	Medical costs for PPI	$163 ($194)	Medical		USD	2019
Pandolfino et al., 2020	pH test	$637 ($759)	Diagnostic tool		USD	2019
	Bravo pH test	$938 ($1,118)				
	Impedance test	$414 ($493)				
Park et al., 2020	Fundoplication	$4,631 ($5,452)	Fundoplication		USD	2020
Azzam et al., 2021	Capsule	$411.53 ($463)	Diagnostic tool		USD	2021
	Catheter	$39.22 ($44)				
Park et al., 2013	Omeprazole 10 mg	241,755 won ($358)	10 mg/day x 24 weeks		Won	2013
	Rabeprazole 10 mg	287,115 won ($425)				
Yokoya et al., 2019	Vonoprazan	¥36,194 ($402)	20 mg/day x 4 weeks		Yen	2019
	Esomeprazole	¥76,719 ($853)				
	Rabeprazole	¥41,105 ($457)				
Funk et al., 2015	Omeprazole, 20 mg twice daily; 6-month supply	$234 ($300.82)	PPI therapy	$1587.40 ($2,040.20)	USD	2015
			Stretta	$14511.18 ($18,655.14)		
			Nissen	$16433.99 ($21,127)		
			EsophyX	$24143.82 ($31,038.58)		
Miwa et al., 2016	Mean medical cost/month	$266 ($338)			USD	2016
Kleiman et al., 2014	pH monitoring/impedance	$340 ($445)			USD	2013
	Esophageal manometry	$350 ($458)				
Habu, 2019	Vonoprazan 20 mg	¥201,6 ($2.24)	20 mg/day x 4 weeks	¥6 290 ($70)	Yen	2019
	Lansoprazole 30 mg	¥124,8 ($1.39)	30 mg/day x 4	¥4 050 ($45)		
	Doctors office visit and physical examination	¥720 ($8)				
	Endoscopic examination	¥14 500 ($161.24)				
Habu et al., 2021	Vonoprazan 10 mg	¥130,3 ($1.45)	10 mg/day x 4 weeks	¥4 410 ($49.17)	Yen	2021
	Lansoprazole 15 mg	¥57,6 ($0.64)	15 mg/day x 4 weeks	¥2 450 ($27.32)		
	Doctors office visit and physical examination	¥730 ($8.14)				
	Endoscopic examination	¥13,360 ($149)				
Ayazi et al., 2020	Magnetic sphincter augmentation (MSA)	$13,522 ($16116)	Surgery	-	USD	2019
	Nissen fundoplication (LNF)	$13,388 ($15956)	Surgery	-		
Singer & Smith, 2021	Cost of WATS3D adjunctive, for screening	$780 ($918)	BE Diagnostic tool	-	USD	2020
	Cost of surveillance EGD + forceps biopsy	$1,442 ($1 697)	BE Diagnostic tool	-		
Gronnier et al., 2014	Surgery (LF)	€397,5 ($918)	Surgery	-	Euro	2013
Owen et al., 2014	CLF	$7,968 ($10,256)	Surgery	-	USD	2014
	RLF	$10,644 ($13,700)				
	OLF	$12,766 ($16,431)				
Furneri et al., 2019	NHS tariff for esophageal endoscopy (£)	£517 ($975)	Diagnostics	-	Pound	2019
McCarty et al., 2022	Omeprazole 20 mg (per pill)	$ 1.54 ($1.60)	PPI strategy	$ 10,931.49 ($11,381.48)	USD	2022
	Omeprazole 40 mg (per pill)	$ 4,39 ($4.57)	TIF strategy	$ 13,978.63 ($14,554)		
	Barium esophagram	$ 230 ($239.47)	LNF strategy	$ 17,658.47 ($18,385.37)		
Harper et al., 2023	Mean medical cost/patient	£4,801 ($7,386)	RefluxStop	£12,204 ($18,775)	Pound	2023
Swart et al., 2021	Cytosponge screening	£77 ($140)	Diagnostics	-	Pound	2021
Honing et al., 2019	Standard endoscopy	$1,821 ($2210)	Diagnostics	-	USD	2018
Törer & Aytaç, 2017	MII catheter + procedure	$120 + $30 ($152 + $38)	Single-step algorithm using MII catheter	$15,300 ($19,424)	USD	2016
			Two-step MII catheter	$16,890 ($21,443)		
Harper et al., 2024	Device – RefluxStop	CHF 6,700 ($6,828)	RefluxStop	CHF 33,780 ($34,425)	CHF	2024
	Procedure cost – all surgical treatments	CHF 13,998 ($14,265)	Nissen fundoplication	CHF 33,844 ($34,490)		
	Device – MSA	CHF 5,170 ($5,269)	MSA	CHF 42,715 ($43,530)		

#### Economic burden of GERD (direct and indirect costs)

Miwa et al. [21] analyzed the medical costs and incidence rates of GERD. In 2014, the GERD prevalence rate was 3.3% in patients aged between 20 and 59 years. The researchers reported that the incidence of GERD increased, and the associated medical cost was approximately 2.4 times the mean national healthcare cost. The most commonly used medications were PPIs. Howden et al. [22] revealed that patients with refractory GERD symptoms incurred greater healthcare costs per patient per year than patients without refractory GERD symptoms ($26 057 vs. $15 285 [constant $2021]) Additionally, des Varannes et al. [23] evaluated the negative impact of GERD on work productivity and daily activities in 716 French patients. Their results indicated a one-third reduction in mean work productivity and daily activities. In addition, the mean associated indirect cost per patient per week was valued at 313 EUR ($541), resulting from the observed productivity loss.

#### PPI therapies

Miwa et al. [21] evaluated the costs of medications and divided them into inpatient and outpatient costs. Each PPI drug was supplied to each patient for 68,5 days. The most commonly used medications were lansoprazole, rabeprazole, esomeprazole, and omeprazole. In patients aged 20–59 years, the mean medical cost per patient per month was $361, divided into inpatient (JPY 12 700 = $144), outpatient (JPY 13 200 = $149), and prescribed drug costs (JPY 6 000 = $68). McCarty et al. [24], in base case analysis, expressed in constant 2022 dollars, showed that the average cost of TIF 2.0 was $ 13,979 vs. $ 17,658 for LNF and $ 10,931 for a PPI. Compared to the PPI strategy, TIF 2.0 was cost-effective, with an incremental cost of $ 3,047 and incremental effectiveness of 0.29 QALYs, resulting in an ICER of $ 10,423 /QALY gained.

Lai et al. [25] showed that directed medication therapy management performed via phone under expert supervision might lower the cost of PPI therapy. Nine out of 40 GERD patients who accepted the conversion to lower-cost PPIs gained a total cost savings of $40,371 per year or $4,486 per patient annually in constant 2022 dollars. When comparing different PPIs, Park et al., [26] showed that omeprazole and rabeprazole were equivalent with respect to the severity and incidence of reflux symptoms. However, omeprazole 10 mg was superior to rabeprazole 10 mg in terms of cost efficiency of maintenance therapy of GERD symptoms. The mean total cost per patient for 6 months was $184 for omeprazole and $219, respectively, for rabeprazole in constant 2020 dollars.

Sharaiha et al. [27] modeled chemoprevention with PPIs to assess their cost-effectiveness in Barrett’s esophagus. The authors developed a Markov model for a hypothetical cohort of patients with Barrett’s esophagus, with one group using PPIs for chemoprevention and the other using no chemoprevention, with endoscopic surveillance for all treatment arms. The authors assumed a 50% reduction in EAC as a result of PPI chemoprevention treatment, which is a cost-effective strategy compared to no chemoprevention. The administration of PPIs incurred a total of $3,706 per patient (incremental cost compared to no chemoprevention group) and resulted in a gain of 0.32 QALYs for an incremental cost-effectiveness ratio of $12,000/QALY based on 2014 constant dollars.

Habu [28] assessed a novel potassium-competitive acid blocker (P-CAB), vonoprazan. These results indicate that it is more cost-effective than PPIs (lansoprazole). Moreover, the entire therapy lasting for 8 weeks resulted in a significantly lower number of days with medication (65 for P-CAB vs. 114 for PPI) and a cost-effectiveness ratio of 58 ($0.64 P-CAB) vs. 68 ($0.756 PPI) yen/day without esophagitis. In a subsequent study, Habu [28] investigated multiple strategies (maintenance with P-CAB or PPI and intermittent treatment with P-CAB or PPI) using the same medications as that in the previous study while employing the Markov model with health state transitions on a monthly basis. The highest cost-effectiveness was obtained for the intermittent P-CAB strategy (¥31 per day ($0.34) without esophagitis), with the total yearly direct medical cost attributable to this strategy being ¥9,380 ($104). Yokoya et al. [29] compared the costs of various drugs among GERD patients, including vonoprazan, esomeprazole, and rabeprazole, for 5 years in 4-week cycles. The most cost-effective strategy in this regard was vonoprazan, which also increased the QALYs. The costs of vonoprazan, esomeprazole, and rabeprazole were ¥36,194 ($402), ¥76,719 ($853), and ¥41,105 ($457), respectively, over a five-year period.

#### Surgical treatment

Gronnier et al. [30] aimed to compare the postoperative health outcomes and direct healthcare costs in patients undergoing Nissen fundoplication (LF) on an outpatient basis, with a single-day discharge, and in patients undergoing LF as inpatients. Health outcomes were comparable between the two groups, but the estimated direct healthcare costs per patient were €2,248 ($5,170) in the day-case group vs. €6,569 ($15,108) in the inpatient group. In addition, Kleppe et al. [31] Evaluated healthcare expenditures after ARS. They reported (in 2020 constant dollars) that the average cost of surgical admission was $24,034. In addition, 4.2% of the patients needed another surgical treatment, which accumulated extra costs of $29,513.

When comparing different types of ARS, the results indicated that some types of ARS were less cost-effective than others, as demonstrated by Schlottmann et al. [32] in a retrospective population-based analysis to compare laparoscopic anti-reflux surgery and open anti-reflux surgery in terms of perioperative outcomes and direct costs. The study included patients diagnosed with GERD (75,544) who underwent either laparoscopic (44,089; 58.4%) or open fundoplication (31,455; 41.6%). According to their findings, direct healthcare cost was reduced by $9,530 in 2017 constant dollars. Similarly, Owen et al. [33] compared data from the US national database to examine perioperative outcomes and costs of open (OF), laparoscopic (CLF), and robotic approaches (RLF) to those of ARS surgery. The data of 12,079 patients showed 2,168 patients underwent OF, 9,572 CLF, and 339 RLF. The results revealed that RLF methods generated equivalent results comparable with those of OF and CLF, with the exception of added cost (mean direct cost of $7,968 for CLF, $10,644 for RLF, and $12,766 for OF in 2014 constant dollars) and a higher readmission rate.

Harper et al. [34] analyzed a novel implantable device, RefluxStop, and found that it showed favorable surgical outcomes compared to both laparoscopic Nissen fundoplication (LNF) and magnetic sphincter augmentation (MSA). The base-case incremental cost-effectiveness ratios compared with MM, LNF, and MSA were £4,156, £6,517, and £249 per QALY gained ($6,393, $10,026, and $383 per QALY), respectively. This analysis has been repeated by the same author in the context of Switzerland’s healthcare system, Markov model was developed using the payer perspective with a lifetime horizon. Findings were similar - ICER for the RefluxStop was CHF 2,116 in comparison to usage of PPI’s. RefluxStop was also found to be cost-effective with probabilities of 97% and 100% against Nissen fundoplication and MSA at a cost-effectiveness threshold of CHF 100,000 per QALY gained [35].

In the following publication, authors analysis aimed to describe the budget impact of introducing RefluxStop within National Health Service (NHS) of and Wales with the development of model adherent to the recommendations of the International Society for Pharmacoeconomics and Outcomes Research with a 5-year time horizon. Introducing RefluxStop alongside currently used PPI, MSA and LNF treatments led to a marginal increase in annual NHS spending on GORD treatment (estimated to be maximally increased by 3,36%) with significant reduction in number of surgical failures, reoperations, and endoscopic dilations [36].

Furthermore, Ayazi et al. [37] evaluated MSA for the treatment of patients with GERD. MSA was compared with standard LNF based on the payer’s reimbursement data (12 months before and after surgery) collected from the database of a local United States insurance company. In total, the data included 195 MSA and 1131 LNF treatments. The median surgery reimbursement was $13,522 for MSA vs. $13,388 for LNF based on 2019 constant dollars. The median reimbursement per month before surgery was $305 for the MSA group vs. $233 for the LNF group, and the postoperative reimbursement per month declined to $104 for MSA vs. $126 for the LNF group. The authors concluded that the MSA results were similar to those for LNF, with a reduction in disease-related expenses for the payer in the year following surgery. However, the surgical costs were slightly higher. Furthermore, Pandolfino et al. [38] predicted cost savings in 2019 constant dollars of $111,367 with the introduction of the MSA (savings of $0.01 per insured member per month).

#### GERD treatment regimen comparisons

Comparisons of different types of treatments indicated that ARS was generally more cost-effective than PPIs in the long term. Park et al. [39] in their Markov model, calculated that surgical therapy would save $551 constant 2016 dollars and increase QALYs by 1.18, compared with the medication group. This finding was also observed in another study by Park et al., [26] which revealed that the costs of medication for surgical intervention significantly decreased compared with those for medication intervention. The most evident difference was among patients aged 20–19 years.

Funk et al. [40] developed a Markov model from the payer’s perspective and found that if PPI treatment costs were higher than $90.63 constant 2020 dollars per month for a period of 30 years, LNF was the preferred therapy choice. Low-cost PPIs, Stretta, and LNF, were shown to be cost-effective treatment strategies.

#### Costs of diagnostic and screening methods for the detection of GERD, Barrett’s esophagus, and adenocarcinoma

As the research suggests, diagnostic and screening methods for the detection of chronic GERD can reduce the costs of treatment with PPIs. Kleiman et al. [41] reported that if the sensitivity of pH monitoring was 96%, early referral for pH monitoring would save between $1,197 and $6,303 of constant 2016 dollars per patient over 10 years. Furthermore, Azzam [42] reported that during reflux monitoring, a wireless system (Bravo) is more user-friendly and generates more benefits for daily activities; however, pH monitoring incurs higher costs. Similarly, Afaneh et al. [43] monitored pH with a BRAVO wireless pH monitoring system to evaluate cost savings compared to empirical PPI therapy. The largest cost savings were observed among patients with extraesophageal symptoms ($2,948–$31,389 of constant 2016 dollars per patient). Lawenko et al. [44] assessed the Bravo system from the viewpoint of cost. The results showed that the full Bravo system price was $25,704, and the cost of one Bravo capsule was $225; meanwhile, the cost of the conventional transnasal pH catheter was $62, all expressed in constant 2016 dollars. Törer et al. [45] compared conventional 24-h pH monitoring with Multichannel Intraluminal Impedance (MII) analysis, which markedly improves the diagnostic accuracy of non-erosive, non-acidic gastro-esophageal reflux disease while being more expensive – $120 compared to $40 of constant 2017 dollars for standard pH catheter. The study authors analyzed medical data from 102 patients in their registry, out of which 36.3% had a Demeester score greater than 14.7. They performed a retrospective projection of costs and used two diagnostic algorithms - one in which the impedance measurement was performed solely and the other following a negative conventional pH monitoring. Based on their study population, the authors concluded that the cost of the single-step algorithm using the MII catheter was $15,300, while the total cost of the two-step scenario was predicted to be $16,890 (constant dollars of 2017). Thus, an approach based solely on a more expensive yet more accurate method, such as the MII analysis, would have been cost-saving.

Several types of screening methods for Barrett’s esophagus are available. However, the most commonly used technique is esophagogastroduodenoscopy with biopsy. Moriarty et al. [46] assessed the direct and indirect costs associated with Barrett’s esophagus screening through a comparative effectiveness randomized trial of unsedated transnasal endoscopy (uTNE, also using a variant of mobile research van [muTNE] instead of hospital [huTNE] variant) and sedated endoscopy (sEGD). Among the 209 patients screened, total costs (direct medical + indirect costs) were higher in the sEGD group ($2022, with $77.76 indirect cost of missed work) than in the uTNE group. The muTNE group had the lowest costs ($286.67; $62.21), followed by the huTNE group ($511; $62.21). Honing et al. [47] used the Markov model to analyze the cost-effectiveness of the new screening modality Endosheath (ultrathin transnasal endoscopy) in a cohort of 50-year-old white men with chronic GERD compared to standard endoscopy and no screening. The study compared the costs of uTNE, standard endoscopy, and no screening at all. The estimated costs were $2,495, $2,957, and $1,436 constant 2018 dollars, respectively. Compared with no screening, uTNE screening resulted in an overall QALY increase of 0.039 and an incremental cost-utility ratio of $29,446 per QALY gained, superior to standard endoscopy – 0.034 QALY and an ICUR of $47,563. Both screening methods seem to be cost-effective, especially when considering a willingness-to-pay cutoff of $50,000.

Moreover, other types of screening for Barrett’s esophagus appear to be cost-effective and have great potential, including narrow-band imaging-guided targeted biopsy [48], wide-area transepithelial sampling with three-dimensional computer-assisted analysis [49], and Cytosponge [50]. However, when comparing different novel screening methods and their tests, Sami et al. [51] showed that the optimal strategy was Cytosponge screening (ICER = $57,500/QALY, based on constant dollars of 2021). In a cost-utility analysis of randomized control data, Swart et al. [52] obtained even more favorable results. By using Markov modeling, they found that one round of Cytosponge-TFF3 screening with confirmatory endoscopy and treatment in the intervention arm costed £82 ($149) more than usual care per person. This intervention generated an additional 0.015 QALYs at an ICER of £5,500 per QALY gained.

Overall, the results showed that the incidence of GERD increased year by year [21], especially among individuals older than 50 years of age. These findings indicated that the most common medical therapies were PPIs, which are cost-effective but may have adverse side effects. Currently, omeprazole is the most cost-effective medication [25]. According to Habu et al. [53] and Yokoya et al. [29], PPIs can be dominated by a novel P-CAB, vonoprazan, with a yearly cost as low as $101 (originally reported in yen), which is related to its higher effectivity and, thus, lower dosage. In addition, the findings indicated that PPIs were dominant in cost-effectiveness comparisons to surgery and endoscopic treatment methods in non-refractory patient cohorts because the PPI treatment per month cost was reported to be as low as $39 (2015 USD) [40], whereas the lowest direct cost of surgery reported was €2,248 ($5,192) [30] in an outpatient mode in France and $10,256 [33] for the United States CLF inpatient variant. This gap will probably widen due to the disadvantage of the ARS after the expansion of vonoprazan use into additional markets. Two studies assessed the novel ARS procedure MSA. However, according to their results, its adoption can lead to only a marginal decrease in surgery costs; thus, it does not change the overall comparison.

To make the ARS competitive with PPIs, the assessment or evaluation period has to be expanded and at least cover 9–30 years with ordinary PPIs [40] (Annex 2) and include refractory GERD patients.

Generally, research on diagnostic tools and screening methods has advanced rapidly, and there remains a question regarding which tools will be the most cost-effective. Therefore, Cytosponge and EsophaCap may play important roles in the detection of Barrett’s esophagus. In addition, narrow-band imaging-guided targeted biopsy [48] and wide-area transepithelial sampling with three-dimensional computer-assisted analysis [49] may enhance biopsy precision in the detection of Barrett’s esophagus, in addition to being cost-effective.

## Discussion

This broad review aimed to provide insights for researchers who are considering the introduction of new approaches to the diagnosis and treatment of GERD. The author´s perspectives on the selection of studies were broader, including a purely cost-effective perspective to comparing ICERs/QALYs and a societal perspective. Thus, this review included studies highlighting the costs of diagnosis, treatment, and care. In addition, the authors attempted to detect GERD-related indirect costs.

The results revealed that there was considerable effort to prevent GERD and Barrett’s through the introduction of new diagnostic tools and screening methods, thereby reducing the overall economic impact associated with treatment, hospitalization, and increasing the QoL of patients with GERD. Miwa et al. [21] explained that in Japan, the mean medical cost per patient with GERD per month was JPY 31,900 ($361), which was about 2,4 times the mean national healthcare cost. In addition, des Varannes et al. [23] stated that the mean indirect cost per patient with GERD per week is 313 EUR ($723), resulting from the observed productivity loss.

These findings show that PPIs remain the gold standard for the treatment of GERD; however, their long-term cost-effectiveness has been questioned [26, 40, 54], suggesting that, in the long run, surgical treatment strategies might be more cost-effective. Funk et al. [40] showed that if the cost of PPI therapy topped $90.63 ($2020) a month over 30 years, LNF would be the preferred treatment option. However, this is valid only assuming long surgery durability of over a decade. In addition, it is necessary to note that most studies in this review reported a monthly cost of PPI therapy that was comfortably below the $90.63 monthly threshold. Furthermore, Kleppe et al. [31] showed that post-surgery readmission was present in 4.2% of patients, and of those, 26.3% might require surgical intervention, leading to an additional cost of up to $34,746. It appears highly improbable for ARS treatment to dominate the newly introduced drug vonoprazan (monthly cost as low as $16 [28]). Ultimately, ARS and endoscopic treatments might be relevant only for patients with serious GERD symptoms for whom PPI or vonoprazan treatment might not be appropriate or for medication non-responders. These findings are consistent with those of a recent review by Jamshed et al. [55] which showed that on-demand (medicine administered after symptom recurrence) treatment with PPIs may have an ICER in 2020 constant dollars as low as $2,197/QALY and was the most effective and cost-saving option compared with all other treatments (i.e., antacids, histamine-2 receptor antagonists, and ARS). However, this seems to contradict the conclusions of recent studies. Park et al. [54] reported that surgical treatment might have cost savings of $551, and the QALYs might improve by 1,18 compared with medical therapy. However, the target cohort in the aforementioned study included patients with severe GERD who required continuous double-dose PPIs. Gockel et al. [56] reached an opposite conclusion, stating that laparoscopic fundoplication seemed to be more cost-effective than long-term medical therapy. However, their findings were based on only six previous studies from 2015, 2013, 2011, 2008, 2004, and 2002. Thus, the review could not describe the shift from branded medication to off-patent generics, which led to significant cost reductions for PPIs, thereby making its conclusions irrelevant to current conditions. Gawron et al. [5] determined that endoscopic anti-reflux procedures are not cost-effective methods and that surgery can be cost-effective compared to medical therapy over a period modeled from 3 years to a lifetime in patients with chronic GERD symptoms.

Recently, wireless esophageal pH monitoring systems for diagnosing GERD and Barrett’s esophagus have become popular. Regarding these new diagnostic approaches, new findings show that the costs of PPI therapy became equivalent to those of the pH monitoring system after 6,4–23,7 weeks, depending on the PPI regimen (Kleiman et al., 2014 [41]). In addition, this wireless pH monitoring system (Bravo) is feasible and safe for monitoring GERD [57]. Besides, they are also well accepted by young adolescents, as shown by Karjoo et al. [58]. However, they are more expensive than conventional catheters [42–44]. Lawenko et al. [44] calculated that a complete Bravo system costs approximately $25,704 constant 2016 dollars. A single-use Bravo capsule with a delivery device costs $225 compared to the conventional trans-nasal pH catheter, which costs $62 in 2018 constant dollars. However, as Afaneh et al. [43] claimed, Bravo wireless pH testing is more cost-effective than prolonged empiric medical management for GERD and should be incorporated early into the treatment algorithm.

Rubenstein et al. [59] stated that conventional esophagogastroduodenoscopy for screening Barrett’s esophagus can prevent 60% of cancer deaths at a cost of $11,254 per QALY gained compared with no screening. Similarly, Honing et al. [47] concluded that Endosheath ultrathin transnasal endoscopy is a cost-effective screening method for Barrett’s esophagus in older white males experiencing GERD symptoms. They reported a cost of $29,446 per QALY gained.

These results indicate that new diagnostic methods are reliable and safe, and many patients judge them to be more comfortable than standard procedures. However, they are often still expensive when compared with other methods, especially in the short run.

In summary, the findings of this review revealed that PPIs appear to be the preferred GERD treatment option in terms of cost-effectiveness. This might change with the adoption of the novel drug vonoprazan, which is more effective and cheaper to use. Furthermore, our results suggest that from a cost-effectiveness point of view, only patients with refractory GERD or patients with serious symptoms should undergo surgery, preferably magnetic sphincter augmentation or laparoscopic fundoplication, where the cost differences are marginal. In addition, with advancements in emerging technologies, more frequent use of new diagnostic and screening approaches should be considered because they might be less invasive and may enhance patients´ QoL while being cost-effective, leading to a change in the entire GERD treatment model.

A significant limitation of this review is the variation in the economic data obtained. The studies included in this review originated predominantly in the USA but also in Europe, Japan, and South Korea. Moreover, the studies were published in different years, and some, typically retrospective studies, used older data that exceeded the period defined for this review. To obtain roughly comparable data, it was necessary to carefully project inflation in the country of publication to 2023 and then prices were converted to USD using purchasing power parity-corrected exchange rates. However, this represents only an approximate approach. The focus of this review also included novel treatments for GERD to describe the treatment progress in the field. Unfortunately, this makes direct comparisons difficult in many instances, such as studies with vonoprazan (a novel P-CAB that dominated PPIs), which originated in Japan, in its domestic market and is currently only under review for its use in the United States by the FDA. Their long-term side effects profile might also be in question, as it was with PPIs, and surfaced only after a significant expansion of their use. In addition, the limited availability of certain treatments and diagnostic methods makes them unsuitable for retrospective studies.

## Conclusions

This review is the first to comprehensively analyze the cost-effectiveness of treatments for GERD, taking into account both direct and indirect costs. The results of this review build on and expand the findings of previous studies [5, 55, 56], indicating that medical care for GERD is becoming increasingly cost-effective. However, the identified studies presented some challenges in comparing estimates due to considerable differences in the time intervals of individual interventions or types of interventions. Future studies on GERD should consider the cost-effectiveness of individual treatment and care approaches in relation to the patient’s QoL.

## Supplementary Information

Supplementary Material 1.

## Data Availability

Data are available on request from the authors.

## References

[1] Arnold M, Abnet CC, Neale RE, et al. Global burden of 5 major types of gastrointestinal Cancer. Gastroenterology. 2020;159(1):335–e34915. 10.1053/j.gastro.2020.02.068. 32247694 10.1053/j.gastro.2020.02.068 PMC8630546

[2] United European Gastroenterology. Burden, economic impact and research gaps: Key findings from the Pan-European study on digestive diseases and cancers. 2024. https://www.nxtbook.com/ueg/UEG/burden-economic-impact-and-research-gaps/index.php#/p/1. Accessed 8 Oct 2024.

[3] Wang R, Li Z, Liu S, Zhang D. Global, regional, and national burden of 10 digestive diseases in 204 countries and territories from 1990 to 2019. Front Public Health. 2023;11:1061453. 10.3389/fpubh.2023.1061453. 37056655 10.3389/fpubh.2023.1061453 PMC10088561

[4] Nirwan JS, Hasan SS, Babar ZUD, Conway BR, Ghori MU. Global prevalence and risk factors of Gastro-oesophageal reflux Disease (GORD): systematic review with Meta-analysis. Sci Rep. 2020;10(1):5814. 10.1038/s41598-020-62795-1. 32242117 10.1038/s41598-020-62795-1 PMC7118109

[5] Gawron AJ, French DD, Pandolfino JE, Howden CW. Economic evaluations of gastroesophageal reflux Disease Medical Management. PharmacoEconomics. 2014;32(8):745–58. 10.1007/s40273-014-0164-8. 24807469 10.1007/s40273-014-0164-8 PMC4394741

[6] Zhang D, Liu S, Li Z, Wang R. Global, regional and national burden of gastroesophageal reflux disease, 1990–2019: update from the GBD 2019 study. Ann Med. 2022;54(1):1372–84. 10.1080/07853890.2022.2074535. 35579516 10.1080/07853890.2022.2074535 PMC9122392

[7] El-Serag HB, Sweet S, Winchester CC, Dent J. Update on the epidemiology of gastro-oesophageal reflux disease: a systematic review. Gut. 2014;63(6):871–80. 10.1136/gutjnl-2012-304269. 23853213 10.1136/gutjnl-2012-304269 PMC4046948

[8] Schwenkglenks M, Marbet UA, Szucs TD. Epidemiology and costs of gastroesophageal reflux disease in Switzerland: a population-based study. Sozial- und Praventivmedizin/Social Prev Medicine. 2004;49(1):51–61. 10.1007/s00038-003-2090-y. 10.1007/s00038-003-2090-y 15040129

[9] Willich SN, Nocon M, Kulig M, et al. Cost-of-disease analysis in patients with gastro-oesophageal reflux disease and Barrett’s mucosa. Aliment Pharmacol Ther. 2006;23(3):371–6. 10.1111/j.1365-2036.2006.02763.x. 16422996 10.1111/j.1365-2036.2006.02763.x

[10] Kamangar F, Nasrollahzadeh D, Safiri S, et al. The global, regional, and national burden of oesophageal cancer and its attributable risk factors in 195 countries and territories, 1990–2017: a systematic analysis for the global burden of Disease Study 2017. The Lancet Gastroenterology Hepatology. 2020;5(6):582–97. 10.1016/S2468-1253(20)30007-8. 32246941 10.1016/S2468-1253(20)30007-8 PMC7232026

[11] Foroutan M, Zojaji H, Ehsani MJ, Darvishi M. Advances in the diagnosis of GERD using the esophageal pH monitoring, gastro-esophageal Impedance-pH monitoring, and Pitfalls. Open Access Maced J Med Sci. 2018;6(10):1934–40. 10.3889/oamjms.2018.410. 30455777 10.3889/oamjms.2018.410 PMC6236049

[12] Kuipers EJ. Barrett esophagus and life expectancy: implications for screening? Gastroenterol Hepatol (N Y). 2011;7(10):689–91. 22298963 PMC3265012

[13] Moher D, Liberati A, Tetzlaff J, Altman DG, The PRISMA Group. Preferred reporting items for systematic reviews and Meta-analyses: the PRISMA Statement. PLoS Med. 2009;6(7):e1000097. 10.1371/journal.pmed.1000097. 19621072 10.1371/journal.pmed.1000097 PMC2707599

[14] Leung XY, Sun J, Bai B. Bibliometrics of social media research: a co-citation and co-word analysis. Int J Hospitality Manage. 2017;66:35–45. 10.1016/j.ijhm.2017.06.012.

[15] Bank of England. Inflation calculator. 2024. https://www.bankofengland.co.uk/monetary-policy/inflation/inflation-calculator. Accessed 8 Oct 2024.

[16] Inflation calculator. 2024. https://tools.csb.gov.lv/cpi_calculator/en/. Accessed 8 Oct 2024.

[17] OECD Data Explorer • OECD Data Archive. OECD. 2024. https://data-explorer.oecd.org/vis?lc=en&df[ds]=DisseminateArchiveDMZ&df[id]=DF_DP_LIVE&df[ag]=OECD&av=true&pd=2022%2C2022&dq=EU27_2020%2BITA%2BAUS%2BAUT%2BBEL%2BCAN%2BCHL%2BCOL%2BCRI%2BCZE%2BDNK%2BEST%2BFIN%2BFRA%2BDEU%2BGRC%2BHUN%2BISL%2BIRL%2BISR%2BJPN%2BKOR%2BLVA%2BLTU%2BLUX%2BMEX%2BNLD%2BNZL%2BNOR%2BPOL%2BPRT%2BSVK%2BSVN%2BESP%2BSWE%2BCHE%2BTUR%2BGBR%2BUSA%2BOAVG%2BOECD.PPP...A&to[TIME_PERIOD]=false&vw=tb&lb=bt. Accessed 8 Oct 2024.

[18] Lata T, Trautman J, Townend P, Wilson RB. Current management of gastro-oesophageal reflux disease—treatment costs, safety profile, and effectiveness: a narrative review. Gastroenterol Rep. 2022;11:goad008. 10.1093/gastro/goad008. 10.1093/gastro/goad008 PMC10112961 37082451

[19] Mathews SC, Izmailyan S, Brito FA, Yamal JM, Mikhail O, Revere FL. Prevalence and Financial Burden of Digestive Diseases in a commercially insured Population. Clin Gastroenterol Hepatol. 2022;20(7):1480–e14877. 10.1016/j.cgh.2021.06.047. 34217877 10.1016/j.cgh.2021.06.047

[20] Katz PO, Dunbar KB, Schnoll-Sussman FH, Greer KB, Yadlapati R, Spechler SJ. ACG Clinical Guideline for the diagnosis and management of gastroesophageal reflux disease. Am J Gastroenterol. 2022;117(1):27–56. 10.14309/ajg.0000000000001538. 34807007 10.14309/ajg.0000000000001538 PMC8754510

[21] Miwa H, Takeshima T, Iwasaki K, Hiroi S. Medical cost, incidence rate, and treatment status of gastroesophageal reflux disease in Japan: analysis of claims data. J Med Econ. 2016;19(11):1049–55. 10.1080/13696998.2016.1192551. 27207316 10.1080/13696998.2016.1192551

[22] Howden CW, Manuel M, Taylor D, Jariwala-Parikh K, Tkacz J. Estimate of Refractory Reflux Disease in the United States: Economic Burden and Associated Clinical characteristics. J Clin Gastroenterol. 2021;55(10):842–50. 10.1097/MCG.0000000000001518. 33780218 10.1097/MCG.0000000000001518

[23] Bruley des Varannes S, Ducrotté P, Vallot T, et al. Gastroesophageal reflux disease: impact on work productivity and daily-life activities of daytime workers. A French cross-sectional study. Dig Liver Disease. 2013;45(3):200–6. 10.1016/j.dld.2012.11.005. 10.1016/j.dld.2012.11.005 23238032

[24] McCarty TR, Jirapinyo P, James LP, Gupta S, Chan WW, Thompson CC. Transoral incisionless fundoplication is cost-effective for treatment of gastroesophageal reflux disease. Endosc Int Open. 2022;10(07):E923–32. 10.1055/a-1783-9378. 35845021 10.1055/a-1783-9378 PMC9286770

[25] Lai L, Alvarez G, Aleu A, Apping C. Cost Avoidance Analysis of Medication Conversions on the Treatment of Gastroesophageal Reflux Disease in a medication Therapy Management Call Center: a budgetary perspective. J Pharm Pract. 2022;35(3):377–82. 10.1177/0897190020977764. 33317384 10.1177/0897190020977764

[26] Park S, Park S, Park JM, et al. Anti-reflux surgery Versus Proton Pump inhibitors for severe gastroesophageal reflux disease: a cost-effectiveness study in Korea. J Neurogastroenterol Motil. 2020;26(2):215–23. 10.5056/jnm19188. 32235028 10.5056/jnm19188 PMC7176505

[27] Sharaiha RZ, Freedberg DE, Abrams JA, Wang YC. Cost-effectiveness of Chemoprevention with Proton Pump inhibitors in Barrett’s Esophagus. Dig Dis Sci. 2014;59(6):1222–30. 10.1007/s10620-014-3186-3. 24795040 10.1007/s10620-014-3186-3 PMC4315516

[28] Habu Y. Vonoprazan versus Lansoprazole for the Initial Treatment of Reflux Esophagitis: a cost-effectiveness analysis in Japan. Intern Med. 2019;58(17):2427–33. 10.2169/internalmedicine.2535-18. 31178490 10.2169/internalmedicine.2535-18 PMC6761357

[29] Yokoya Y, Igarashi A, Uda A, Deguchi H, Takeuchi T, Higuchi K. Cost-utility analysis of a ‘vonoprazan-first’ strategy versus ‘esomeprazole- or rabeprazole-first’ strategy in GERD. J Gastroenterol. 2019;54(12):1083–95. 10.1007/s00535-019-01609-2. 31396703 10.1007/s00535-019-01609-2

[30] Gronnier C, Desbeaux A, Piessen G, et al. Day-case versus inpatient laparoscopic fundoplication: outcomes, quality of life and cost-analysis. Surg Endosc. 2014;28(7):2159–66. 10.1007/s00464-014-3448-3. 24515264 10.1007/s00464-014-3448-3

[31] Kleppe KL, Xu Y, Funk LM, et al. Healthcare spending and utilization following antireflux surgery: examining costs and reasons for readmission. Surg Endosc. 2020;34(1):240–8. 10.1007/s00464-019-06758-2. 30953200 10.1007/s00464-019-06758-2

[32] Schlottmann F, Strassle PD, Patti MG. Comparative Analysis of Perioperative Outcomes and costs between laparoscopic and open antireflux surgery. J Am Coll Surg. 2017;224(3):327–33. 10.1016/j.jamcollsurg.2016.12.010. 28132820 10.1016/j.jamcollsurg.2016.12.010

[33] Owen B, Simorov A, Siref A, Shostrom V, Oleynikov D. How does robotic anti-reflux surgery compare with traditional open and laparoscopic techniques: a cost and outcomes analysis. Surg Endosc. 2014;28(5):1686–90. 10.1007/s00464-013-3372-y. 24414455 10.1007/s00464-013-3372-y

[34] Harper S, Grodzicki L, Mealing S, Gemmill L, Goldsmith PJ, Ahmed AR. Cost-effectiveness of a novel, non-active implantable device as a treatment for refractory gastro-esophageal reflux disease. J Med Econ. 2023;26(1):603–13. 10.1080/13696998.2023.2201063. 37042668 10.1080/13696998.2023.2201063

[35] Harper S, Kartha M, Mealing S, Borbély YM, Zehetner J. Cost-effectiveness of the RefluxStop device for management of refractory gastroesophageal reflux disease in Switzerland. J Med Econ. 2024;27(1):805–15. 10.1080/13696998.2024.2362564. 38820006 10.1080/13696998.2024.2362564

[36] Harper S, Grodzicki L, Mealing S, Gemmill E, Goldsmith P, Ahmed A. Budget Impact of RefluxStop^™^ as a treatment for patients with refractory gastro-oesophageal Reflux Disease in the United Kingdom. J Health Econ Outcomes Res. 2024;11(1). 10.36469/001c.90924. 10.36469/001c.90924 PMC10787539 38222857

[37] Ayazi S, Zaidi AH, Zheng P, et al. Comparison of surgical payer costs and implication on the healthcare expenses between laparoscopic magnetic sphincter augmentation (MSA) and laparoscopic Nissen fundoplication (LNF) in a large healthcare system. Surg Endosc. 2020;34(5):2279–86. 10.1007/s00464-019-07021-4. 31376004 10.1007/s00464-019-07021-4 PMC7113225

[38] Pandolfino J, Lipham J, Chawla A, Ferko N, Hogan A, Qadeer RA. A budget impact analysis of a magnetic sphincter augmentation device for the treatment of medication-refractory mechanical gastroesophageal reflux disease: a United States payer perspective. Surg Endosc. 2020;34(4):1561–72. 10.1007/s00464-019-06916-6. 31559575 10.1007/s00464-019-06916-6

[39] Park S, Kwon JW, Park JM, Park S, Hwang J, Seo KW. The characteristics of antireflux surgery compared to Proton pump inhibitor treatment in Korea: a nationwide study using claim data from 2007 to 2016. Ann Surg Treat Res. 2020;98(5):254. 10.4174/astr.2020.98.5.254. 32411630 10.4174/astr.2020.98.5.254 PMC7200601

[40] Funk LM, Zhang JY, Drosdeck JM, Melvin WS, Walker JP, Perry KA. Long-term cost-effectiveness of medical, endoscopic and surgical management of gastroesophageal reflux disease. Surgery. 2015;157(1):126–36. 10.1016/j.surg.2014.05.027. 25262216 10.1016/j.surg.2014.05.027

[41] Kleiman DA, Beninato T, Bosworth BP, et al. Early Referral for esophageal pH monitoring is more cost-effective than prolonged empiric trials of Proton-Pump inhibitors for suspected gastroesophageal reflux disease. J Gastrointest Surg. 2014;18(1):26–34. 10.1007/s11605-013-2327-x. 24214090 10.1007/s11605-013-2327-x

[42] Azzam RS, Azzam GB, Nasi A, WIRELESS PH MONITORING AND CONVENTIONAL ESOPHAGEAL PH, MONITORING: COMPARATIVE STUDY OF DISCOMFORT, LIMITATIONS IN DAILY ACTIVITIES AND COMPLICATIONS. ABCD arq bras cir dig. 2021;34(1):e1566. 10.1590/0102-672020210001e1566. 34008710 10.1590/0102-672020210001e1566 PMC8121045

[43] Afaneh C, Zoghbi V, Finnerty BM, et al. BRAVO esophageal pH monitoring: more cost-effective than empiric medical therapy for suspected gastroesophageal reflux. Surg Endosc. 2016;30(8):3454–60. 10.1007/s00464-015-4629-4. 26537906 10.1007/s00464-015-4629-4

[44] Lawenko RMA, Lee YY. Evaluation of gastroesophageal reflux Disease using the Bravo Capsule pH system. J Neurogastroenterol Motil. 2015;22(1):25–30. 10.5056/jnm15151. 10.5056/jnm15151 PMC4699719 26717929

[45] Törer N, Aytaç Ö. Is the routine use of Impedance Analysis for the diagnosis of Gastro-Esophageal Reflux Disease more expensive than conventional pH monitoring? Cost analysis of two procedures. Indian J Surg. 2017;79(3):192–5. 10.1007/s12262-016-1444-7. 28659670 10.1007/s12262-016-1444-7 PMC5473786

[46] Moriarty JP, Shah ND, Rubenstein JH, et al. Costs associated with Barrett’s esophagus screening in the community: an economic analysis of a prospective randomized controlled trial of sedated versus hospital unsedated versus mobile community unsedated endoscopy. Gastrointest Endosc. 2018;87(1):88–e942. 10.1016/j.gie.2017.04.019. 28455158 10.1016/j.gie.2017.04.019 PMC5656556

[47] Honing J, Kievit W, Bookelaar J, Peters Y, Iyer PG, Siersema PD. Endosheath ultrathin transnasal endoscopy is a cost-effective method for screening for Barrett’s esophagus in patients with GERD symptoms. Gastrointest Endosc. 2019;89(4):712–e7223. 10.1016/j.gie.2018.10.024. 30385112 10.1016/j.gie.2018.10.024

[48] Furneri G, Klausnitzer R, Haycock L, Ihara Z. Economic value of narrow-band imaging versus white light endoscopy for the diagnosis and surveillance of Barrett’s esophagus: Cost-consequence model. Geisler BP, ed. PLoS ONE. 2019;14(3):e0212916. 10.1371/journal.pone.0212916. 30865673 10.1371/journal.pone.0212916 PMC6415878

[49] Singer ME, Smith MS. Wide area transepithelial sampling with computer-assisted analysis (WATS3D) is cost-effective in Barrett’s Esophagus Screening. Dig Dis Sci. 2021;66(5):1572–9. 10.1007/s10620-020-06412-1. 32578042 10.1007/s10620-020-06412-1 PMC8053177

[50] Benaglia T, Sharples LD, Fitzgerald RC, Lyratzopoulos G. Health benefits and cost effectiveness of endoscopic and nonendoscopic cytosponge screening for Barrett’s Esophagus. Gastroenterology. 2013;144(1):62–e736. 10.1053/j.gastro.2012.09.060. 23041329 10.1053/j.gastro.2012.09.060

[51] Sami SS, Moriarty JP, Rosedahl JK, et al. Comparative cost effectiveness of reflux-based and reflux-independent strategies for Barrett’s Esophagus Screening. Am J Gastroenterol. 2021;116(8):1620–31. 10.14309/ajg.0000000000001336. 34131096 10.14309/ajg.0000000000001336 PMC8315187

[52] Swart N, Maroni R, Muldrew B, Sasieni P, Fitzgerald RC, Morris S. Economic evaluation of Cytosponge^®^-trefoil factor 3 for Barrett esophagus: a cost-utility analysis of randomised controlled trial data. eClinicalMedicine. 2021;37:100969. 10.1016/j.eclinm.2021.100969. 34195582 10.1016/j.eclinm.2021.100969 PMC8225801

[53] Habu Y, Hamasaki R, Maruo M, Nakagawa T, Aono Y, Hachimine D. Treatment strategies for reflux esophagitis including a potassium-competitive acid blocker: a cost‐effectiveness analysis in Japan. J Gen Fam Med. 2021;22(5):237–45. 10.1002/jgf2.429. 34484992 10.1002/jgf2.429 PMC8411401

[54] Park S, Kwon JW, Park JM, Park S, Seo KW. Treatment pattern and economic Burden of Refractory Gastroesophageal Reflux Disease patients in Korea. J Neurogastroenterol Motil. 2020;26(2):281–8. 10.5056/jnm19050. 31682754 10.5056/jnm19050 PMC7176495

[55] Jamshed S, Bhagavathula AS, Zeeshan Qadar SM, Alauddin U, Shamim S, Hasan S. Cost-effective analysis of Proton Pump inhibitors in long-term management of gastroesophageal reflux disease: a narrative review. Hosp Pharm. 2020;55(5):292–305. 10.1177/0018578719893378. 32999499 10.1177/0018578719893378 PMC7502866

[56] Gockel I, Lange UG, Schürmann O, et al. Kosten-Effektivitäts- Und Kosten-Nutzwert-Analysen Der Antirefluxmedizin. Gesundheitswesen. 2019;81(12):1048–56. 10.1055/a-0586-3630. 29649837 10.1055/a-0586-3630

[57] Yang L, Chaudhary N, Baghdadi J, Pei Z. Microbiome in Reflux Disorders and esophageal adenocarcinoma. Cancer J. 2014;20(3):207–10. 10.1097/PPO.0000000000000044. 24855009 10.1097/PPO.0000000000000044 PMC4120752

[58] Karjoo M, Beg M, Kesselring S, Reliability. Safety and Effectiveness of the Bravo(TM) Capsule: a catheter-free pH monitoring system for evaluation of Gastroesophageal Reflux Disease in children. Middle East J Dig Dis. 2012;4(1):34–9. 24829633 PMC4017693

[59] Rubenstein JH, Inadomi JM, Brill JV, Eisen GM. Cost utility of screening for Barrett’s Esophagus with Esophageal Capsule Endoscopy Versus Conventional Upper Endoscopy. Clin Gastroenterol Hepatol. 2007;5(3):312–8. 10.1016/j.cgh.2006.12.008. 17368230 10.1016/j.cgh.2006.12.008

