# Supplementary material for: Diagnosis and treatment of patients with gastroesophageal reflux disease – a systematic review of cost-effectiveness and economic burden
Source: BMC Health Serv Res. 2024 Nov 6;24:1351. doi: 10.1186/s12913-024-11781-8 (PMC11539747; doi:10.1186/s12913-024-11781-8)
Supplement: Supplementary file 1 — Supplementary Material 1. [file 12913_2024_11781_MOESM1_ESM.docx]

**Annex 1:** Overview of included studies

| **Author, Year** | **Article Title** | **Publication Year** | **Number of citations** |
| --- | --- | --- | --- |
| Lawenko, R. M. A., & Lee, Y. Y. (2015) | **Evaluation of Gastroesophageal Reflux Disease Using the Bravo Capsule pH System** | 2016 | 13 |
| Afaneh, C., Zoghbi, V., Finnerty, B. M., Aronova, A., Kleiman, D., Ciecierega, T., Crawford, C., Fahey, T. J., III, & Zarnegar, R. (2015) | **BRAVO esophageal pH monitoring: more cost-effective than empiric medical therapy for suspected gastroesophageal reflux** | 2016 | 6 |
| Sami, S. S., Moriarty, J. P., Rosedahl, J. K., Borah, B. J., Katzka, D. A., Wang, K. K., Kisiel, J. B., Ragunath, K., Rubenstein, J. H., & Iyer, P. G. (2021) | **Comparative Cost Effectiveness of Reflux-Based and Reflux-Independent Strategies for Barrett's Esophagus Screening** | 2021 | 3 |
| Benaglia, T., Sharples, L. D., Fitzgerald, R. C., & Lyratzopoulos, G. (2013) | **Health Benefits and Cost Effectiveness of Endoscopic and Nonendoscopic Cytosponge Screening for Barrett's Esophagus** | 2013 | 111 |
| Park, S., Park, S., Park, J.-M., Ryu, S., Hwang, J., Kwon, J.-W., & Seo, K. W. (2020) | **Anti-reflux Surgery Versus Proton Pump Inhibitors for Severe Gastroesophageal Reflux Disease: A Cost-Effectiveness Study in Korea** | 2020 | 5 |
| Park, S., Kwon, J.-W., Park, J.-M., Park, S., & Seo, K. W. (2020) | **Treatment Pattern and Economic Burden of Refractory Gastroesophageal Reflux Disease Patients in Korea** | 2020 | 8 |
| Pandolfino, J., Lipham, J., Chawla, A., Ferko, N., Hogan, A., & Qadeer, R. A. (2019) | **A budget impact analysis of a magnetic sphincter augmentation device for the treatment of medication-refractory mechanical gastroesophageal reflux disease: a United States payer perspective** | 2019 | 3 |
| Park, S., Kwon, J.-W., Park, J.-M., Park, S., Hwang, J., & Seo, K. W. (2020) | **The characteristics of antireflux surgery compared to proton pump inhibitor treatment in Korea: a nationwide study using claim data from 2007 to 2016** | 2020 | 4 |
| AZZAM, R. S., AZZAM, G. B., & NASI, A. (2021) | **WIRELESS PH MONITORING AND CONVENTIONAL ESOPHAGEAL PH MONITORING: COMPARATIVE STUDY OF DISCOMFORT, LIMITATIONS IN DAILY ACTIVITIES AND COMPLICATIONS** | 2021 | 6 |
| Park, J. H., Park, H., Lee, D. H., & Sung, I. K. (2013) | **A Randomized, Double Blinded, Clinical Trial to Assess the Efficacy and Cost Effectiveness of Omeprazole Compared to Rabeprazole in the Maintenance Therapy of Patients With Gastroesophageal Reflux Disease** | 2013 | 2 |
| Yokoya, Y., Igarashi, A., Uda, A., Deguchi, H., Takeuchi, T., & Higuchi, K. (2019) | **Cost-utility analysis of a 'vonoprazan-first' strategy versus 'esomeprazole- or rabeprazole-first' strategy in GERD** | 2019 | 7 |
| Funk, L. M., Zhang, J. Y., Drosdeck, J. M., Melvin, W. S., Walker, J. P., & Perry, K. A. (2015) | **Long-term cost-effectiveness of medical, endoscopic and surgical management of gastroesophageal reflux disease** | 2015 | 30 |
| Heberle, C. R., Omidvari, A.-H., Ali, A., Kroep, S., Kong, C. Y., Inadomi, J. M., Rubenstein, J. H., Tramontano, A. C., Dowling, E. C., Hazelton, W. D., Luebeck, E. G., Lansdorp-Vogelaar, I., & Hur, C. (2017) | **Cost Effectiveness of Screening Patients With Gastroesophageal Reflux Disease for Barrett's Esophagus With a Minimally Invasive Cell Sampling Device** | 2017 | 25 |
| Miwa, H., Takeshima, T., Iwasaki, K., & Hiroi, S. (2016) | **Medical cost, incidence rate, and treatment status of gastroesophageal reflux disease in Japan: analysis of claims data** | 2016 | 7 |
| Kleiman, D. A., Beninato, T., Bosworth, B. P., Brunaud, L., Ciecierega, T., Crawford, C. V., Jr, Turner, B. G., Fahey, T. J., III, & Zarnegar, R. (2013) | **Early Referral for Esophageal pH Monitoring Is More Cost-Effective Than Prolonged Empiric Trials of Proton-Pump Inhibitors for Suspected Gastroesophageal Reflux Disease** | 2013 | 6 |
| Habu, Y. (2019) | **Vonoprazan versus Lansoprazole for the Initial Treatment of Reflux Esophagitis: A Cost-effectiveness Analysis in Japan** | 2019 | 5 |
| Habu, Y., Hamasaki, R., Maruo, M., Nakagawa, T., Aono, Y., & Hachimine, D. (2021) | **Treatment strategies for reflux esophagitis including a potassium-competitive acid blocker: A cost-effectiveness analysis in Japan** | 2021 | 3 |
| Bruley des Varannes, S., Ducrotté, P., Vallot, T., Garofano, A., Bardoulat, I., Carrois, F., & Ricci, L. (2013) | **Gastroesophageal reflux disease: Impact on work productivity and daily-life activities of daytime workers. A French cross-sectional study** | 2013 | 7 |
| Howden, C. W., Manuel, M., Taylor, D., Jariwala-Parikh, K., & Tkacz, J. (2021) | **Estimate of Refractory Reflux Disease in the United States Economic Burden and Associated Clinical Characteristics** | 2021 | 1 |
| Ayazi, S., Zaidi, A. H., Zheng, P., Chovanec, K., Chowdhury, N., Salvitti, M., Newhams, K., Levy, J., Hoppo, T., & Jobe, B. A. (2020) | **Comparison of surgical payer costs and implication on the healthcare expenses between laparoscopic magnetic sphincter augmentation (MSA) and laparoscopic Nissen fundoplication (LNF) in a large healthcare system** | 2020 | 4 |
| Singer, M. E., & Smith, M. S. (2021) | **Wide Area Transepithelial Sampling with Computer-Assisted Analysis (WATS(3D)) Is Cost-Effective in Barrett's Esophagus Screening** | 2021 | 5 |
| Gronnier, C., Desbeaux, A., Piessen, G., Boutillier, J., Ruolt, N., Triboulet, J. P., & Mariette, C. (2014) | **Day-case versus inpatient laparoscopic fundoplication: outcomes, quality of life and cost-analysis** | 2014 | 16 |
| Lai, L., Alvarez, G., Aleu, A., & Apping, C. (2020) | **Cost Avoidance Analysis of Medication Conversions on the Treatment of Gastroesophageal Reflux Disease in a Medication Therapy Management Call Center: A Budgetary Perspective** | 2022 | 0 |
| Kleppe, K. L., Xu, Y., Funk, L. M., Wang, X., Havlena, J. A., Greenberg, J. A., & Lidor, A. O. (2019) | **Healthcare spending and utilization following antireflux surgery: examining costs and reasons for readmission** | 2020 | 4 |
| Sharaiha, R. Z., Freedberg, D. E., Abrams, J. A., & Wang, Y. C. (2014) | **Cost-Effectiveness of Chemoprevention with Proton Pump Inhibitors in Barrett's Esophagus** | 2014 | 7 |
| Owen, B., Simorov, A., Siref, A., Shostrom, V., & Oleynikov, D. (2014) | **How does robotic anti-reflux surgery compare with traditional open and laparoscopic techniques: a cost and outcomes analysis** | 2014 | 26 |
| Schlottmann, F., Strassle, P. D., & Patti, M. G. (2017) | **Comparative Analysis of Perioperative Outcomes and Costs Between Laparoscopic and Open Antireflux Surgery** | 2017 | 14 |
| Furneri, G., Klausnitzer, R., Haycock, L., & Ihara, Z. (2019) | **The economic value of narrow-band imaging versus white light endoscopy for the diagnosis and surveillance of Barrett's esophagus: Cost-consequence model** | 2019 | 4 |
| Yang Y., Chen H. N., Wang R., Tang Y. J., Chen X. Z. (2015) | **Cost-Effectiveness Analysis on Endoscopic Surveillance Among Western Patients With Barrett's Esophagus for Esophageal Adenocarcinoma Screening** | 2015 | 1 |
| Moriarty, J. P., Shah, N. D., Rubenstein, J. H., Blevins, C. H., Johnson, M., Katzka, D. A., Wang, K. K., Wongkeesong, L. M., Ahlquist, D. A., & Iyer, P. G. (2018) | **Costs associated with Barrett's esophagus screening in the community: an economic analysis of a prospective randomized controlled trial of sedated versus hospital unsedated versus mobile community unsedated endoscopy** | 2018 | 21 |
| McCarty, T. R., Jirapinyo, P., James, L. P., Gupta, S., Chan, W. W., & Thompson, C. C. (2022) | **Transoral incisionless fundoplication is cost-effective for treatment of gastroesophageal reflux disease** | 2022 | 5 |
| Harper, S., Grodzicki, L., Mealing, S., Gemmill, L., Goldsmith, P. J., & Ahmed, A. R. (2023) | **Cost-effectiveness of a novel, non-active implantable device as a treatment for refractory gastro-esophageal reflux disease** | 2023 | 4 |
| Swart, N., Maroni, R., Muldrew, B., Sasieni, P., Fitzgerald, R. C., & Morris, S. (2021) | **Economic evaluation of Cytosponge®-trefoil factor 3 for Barrett esophagus: A cost-utility analysis of randomised controlled trial data** | 2021 | 7 |
| Honing, J., Kievit, W., Bookelaar, J., Peters, Y., Iyer, P. G., & Siersema, P. D. (2019) | **Endosheath ultrathin transnasal endoscopy is a cost-effective method for screening for Barrett’s esophagus in patients with GERD symptoms** | 2019 | 17 |
| Törer, N., & Aytaç, Ö. (2017) | **Is the Routine Use of Impedance Analysis for the Diagnosis of Gastro-Esophageal Reflux Disease More Expensive than Conventional pH Monitoring? Cost Analysis of Two Procedures** | 2017 | 1 |
| Harper, S., Kartha, M., Mealing, S., Borbély, Y. M., & Zehetner, J. (2024) | **Cost-effectiveness of the RefluxStop device for management of refractory gastroesophageal reflux disease in Switzerland** | 2024 | 0 |

**Annex 2**: Study characteristics of the concomitant health economic evaluation

| Study | Modelling approaches  / model type | Number and Type of health conditions / events | Cycle length (when using Markov models) /  timeline | Assumptions | Handling of uncertainty / statements / assertions regarding model validation |
| --- | --- | --- | --- | --- | --- |
| Afaneh et al., 2016 | Cost model, Cost equivalence calculation | Esophagogastroduodenoscopy, pH monitoring, Esophageal manometry | Median PPI use prior to referral was 215 weeks (range  0–520) | The records of 100 consecutive patients who underwent  BRAVO esophageal pH monitoring for suspected GERD  on PPI therapy | The study selected for patients with  symptomatic GERD on PPI therapy who were offered pH  testing, and thus, not all patients with symptomatic GERD  were captured. |
| Sami et al., 2021 | Markov model | sedated endoscopy (sEGD), transnasal endoscopy | 1-month cycle length | Various types in model (BE, NDBE, LGD, HGD, IMC/SC) + combinations | One-way sensitivity analyses |
| Benaglia et al., 2013 | Microsimulation modeling | 50-year-old men with a history of symptoms of gastroesophageal reflux disease (GERD) | Duration of the microsimulation cycle (30  days) | Cytosponge or endoscopy  screening | Supplementary and sensitivity analyses comprised |
| Park et al., 2020 | Markov model | Surgery, medical treatment | 10 years | It targeted the parameter assumptions  that varied ± 25% from both the surgical and medical strategies  in the baseline case model. | Sensitivity analyses using the varying parameter assumptions demonstrated the robustness of the study results. |
| Park et al., 2020 | Generalized linear models | Surgery | 4 years | PPIs medication for more than 12  weeks | Combination of the univariate and multivariable model |
| Pandolfino et al., 2020 | Economic budget impact model | 2 surgery treatment types (MSA vs LNF) | developed over a 1-year time horizon | MSA patients experiencing  device removal reoperations were assumed to not require  additional treatments | Real-world scenarios underlying this study |
| Yokoya et al., 2019 | Markov simulation model | Cost-effectiveness of the vonoprazan-first ‘top-down’ dosing strategy for symptomatic GERD versus two other PPI first ‘step-up’ strategies for esomeprazole and rabeprazole. | Comprising healing and maintenance therapies, over 5 years (4-  week cycles). | The base treatment strategy comprised 4 weeks of healing therapy at a dose of 20 mg once daily and, for healed patients, 6 months of maintenance treatment at a dose of 10 mg once daily. | A one-way sensitivity analysis to assess the impact of  variations in key parameters on incremental costs and  QALYs was performed |
| Funk et al., 2015 | Markov model | PPI therapy, transoral incisionless  fundoplication (EsophyX), radiofrequency energy application to the lower esophageal sphincter (Stretta)  and laparoscopic Nissen fundoplication | 6-month cycle and 30-year  time horizon | Symptomatic GERD taking 20 mg of  omeprazole twice daily | Additionally,  was performed 1-way sensitivity analyses to determine  how varying costs and relapse rates impacted the  cost effectiveness of each treatment modality |
| Heberle et al., 2017 | Two  validated microsimulation models | Endoscopy alone or cytosponge | Every year for low-grade dysplasia, every three years for non-dysplastic  BE | BE in male patients with GERD, 60 years  of age | One-way sensitivity analyses on several key parameters, including cytosponge cost,  cytosponge performance characteristics, initial effectiveness of EET, rates of recurrence after EET,  gender, and the age of initial screening. |
| Kleiman et al., 2014 | A cost model | Esophageal  GERD symptoms | 7 years | Underwent 24-h esophageal pH monitoring | Two-way sensitivity  analysis was performed to test the cost model over the range  of possible costs |
| Habu, 2019 | Markov model | No. of days without esophagitis | 1 year | Healing rate (4 weeks): Vonoprazan (20mg / day) 0.966; Lansoprazole (30mg/day) 0.925; Relapse after healing 0.14/month; Endoscopy to confirm healing 0.145 | Probabilistic, Probability of healing with given treatment varied, probability of necessity to perform endoscopy varied |
| Habu et al., 2021 | Markov model | No. of days without esophagitis | 1 year | Healing rate (4 weeks): Vonoprazan (20mg / day) 0.966; Lansoprazole (30mg/day) 0.925; Relapse after healing 0.14/month; Endoscopy to confirm healing 0(0-1) | Probabilistic, Probability of healing with given treatment varied, probability of necessity to perform endoscopy varied |
| Bruley des Varannes et al., 2013 | - | Mean work  productivity and daily activities decrease | 1 year | Questionnaire | - |
| Howden et al., 2021 | - | Cost of care for GERD patients w/o refractory symptoms | 1 year | - | - |
| Ayazi et al., 2020 | - | 2 surgery treatment types (MSA vs LNF) | 2 year | - | - |
| Singer & Smith, 2021 | A decision analytic model | Screening for BE with random 4-quadrant forceps biopsies (FB) and FB plus WATS 3D | - | Added Yield of WATS3D (an increase of BE detection) 71-213%; false positive rate of WATS3D 5-25% | One-way sensitivity analysis was performed on all model  parameters. Two-way sensitivity analysis was performed on  WATS3D parameters  . |
| Gronnier et al., 2014 | - | Surgery (LF) | 2 years (last patient assessment after surgery) | - | - |
| Lai et al., 2022 | Extrapolation (of medication conversion to lower-cost PPIs) | - | 1 year | Successful medication conversion to lower-cost PPIs | None (on per patient level) |
| Kleppe et al., 2020 | - | ARS surgery, repeated ARS surgery | 90 days | - | - |
| Sharaiha et al., 2014 | Markov model | the prevention of EAC in Barrett’s esophagus without reflux | 30-years | 50 % reduction in esophageal adenocarcinoma (EAC) occurrence with PPI therapy | Reduction in EAC occurrence with PPI therapy varied (0-1), and treatment costs varied (base, low, high estimates) |
| Owen et al., 2014 | - | Surgery (LF) | - | - | - |
| Schlottmann et al., 2017 | - | Surgery (LF) | - | - | - |
| Furneri et al., 2019 | decision tree / Markov model | Diagnostics, Esophegal endoscopy + biopsy | 7 years | NBI targeted biopsies have the same detection rate as an HD-WLE examination with the Seattle protocol but require fewer biopsies (3.6 vs 7.6 respectively). Per-location sensitivity and specificity for the detection of BE - sensitivity: HD-WLE = 79.1%; NBI = 89.0%; ; Specificity: HD-WLE = 81.0%; NBI = 80.0%; | N of biopsies per intervention varied (+/-10%); Cost per biopsy (+/-10%); N of biopsies per intervention (+/-10%) |
| Yang et al., 2015 | Cost-effectiveness decision tree | EAC death | 5 years |  | Sensitivity analysis – the varying percentage of participants in adequate surveillance group 0-0.9 |
| Moriarty et al., 2018 | - | Diagnostics, transnasal endoscopy | 30 days | BE prevalence in population = 1,6%; patients participation in screening modes: AS=57,14%; IAS=15,7%; NS=27,69%; | - |
| McCarty et al., 2022 | Markov cohort model | Surgery, medical treatment | 10-year time horizon and cycle length of 12 months | Refractory GERD symptoms despite twice-daily PPI therapy | One-way and probabilistic sensitivity analyses were also performed to explore the uncertainty around model input parameters |
| Harper et al., 2023 | Markov model | Surgery, medical treatment | 30 days | Patients in the model were at risk of developing Barrett’s esophagus | Deterministic sensitivity analyses were conducted to assess the inherent uncertainty associated with model results |
| Swart et al., 2021 | Markov model | Cytosponge screening | One-year cycle-length and a lifetime time horizon | Cytosponge-TFF3 screening | Probabilistic sensitivity analysis (PSA) and deterministic sensitivity analysis (DSA) |
| Honing et al., 2019 | Markov model | standard endoscopy | Lifetime horizon | Chronic GERD symptoms | Sensitivity analyses were performed to demonstrate differences in outcome by using a minimum and maximum value for several variables |
| Harper et al., 2024 | Markov model | Surgery, medical treatment | 30 days | All patients were presumed to be managed with PPIs as a first-line therapy | Deterministic sensitivity analyses were conducted to assess the inherent uncertainty associated with model results |

**Annex 3**. Article filtering procedure

| **Key words used “AND” between all words** | **WOS** | **PubMed** |
| --- | --- | --- |
| Gastroesophageal Reflux Disease AND Technology | 962 | 910 |
| Gastroesophageal Reflux Disease AND Cost | 453 | 470 |
| Gastroesophageal Reflux Disease AND Capsule | 299 | 112 |
| Gastroesophageal Reflux Disease AND Technology AND Cost | 65 | 52 |
| Gastroesophageal Reflux Disease AND Capsule AND Cost | 39 | 8 |
| Proton Pump Inhibitors AND Cost | 695 | 525 |
| Proton Pump Inhibitors AND Cost AND Reflux | 192 | 154 |
| **Total** | **2,705** | **2,231** |
